# Proteomic analysis reveals key proteins involved in ethylene-induced adventitious root development in cucumber (*Cucumis sativus* L.)

**DOI:** 10.7717/peerj.10887

**Published:** 2021-04-06

**Authors:** Jian Lyu, Yue Wu, Xin Jin, Zhongqi Tang, Weibiao Liao, Mohammed Mujitaba Dawuda, Linli Hu, Jianming Xie, Jihua Yu, Alejandro Calderón-Urrea

**Affiliations:** 1 College of Horticulture, Gansu Agricultural University, Lanzhou, China; 2 College of Agriculture and Forestry Science, Linyi University, Linyi, China; 3 Department of Horticulture, University for Development Studies, Tamale, Ghana; 4 Gansu Provincial Key Laboratory of Arid Land Crop Science, Gansu Agricultural University, Lanzhou, China; 5 Department of Biology, College of Science and Mathematics, California State University, CA, USA; 6 College of Plant Protection, Gansu Agricultural University, Lanzhou, China

**Keywords:** Ethylene, Adventitious root, Proteomics, Ethylene synthesis, Photosynthetic carbon assimilation

## Abstract

The mechanisms involved in adventitious root formation reflect the adaptability of plants to the environment. Moreover, the rooting process is regulated by endogenous hormone signals. Ethylene, a signaling hormone molecule, has been shown to play an essential role in the process of root development. In the present study, in order to explore the relationship between the ethylene-induced adventitious rooting process and photosynthesis and energy metabolism, the iTRAQ technique and proteomic analysis were employed to ascertain the expression of different proteins that occur during adventitious rooting in cucumber (*Cucumis sativus* L.) seedlings. Out of the 5,014 differentially expressed proteins (DEPs), there were 115 identified DEPs, among which 24 were considered related to adventitious root development. Most of the identified proteins were related to carbon and energy metabolism, photosynthesis, transcription, translation and amino acid metabolism. Subsequently, we focused on S-adenosylmethionine synthase (SAMS) and ATP synthase subunit a (AtpA). Our findings suggest that the key enzyme, SAMS, upstream of ethylene synthesis, is directly involved in adventitious root development in cucumber. Meanwhile, AtpA may be positively correlated with photosynthetic capacity during adventitious root development. Moreover, endogenous ethylene synthesis, photosynthesis, carbon assimilation capacity, and energy material metabolism were enhanced by exogenous ethylene application during adventitious rooting. In conclusion, endogenous ethylene synthesis can be improved by exogenous ethylene additions to stimulate the induction and formation of adventitious roots. Moreover, photosynthesis and starch degradation were enhanced by ethylene treatment to provide more energy and carbon sources for the rooting process.

## Introduction

The rooting process of adventitious roots is a crucial growth process in plants. The efficiency of adventitious rooting reflects the adaptability of plants to various environmental conditions, such as waterlogging, nutrient deficiency, and mechanical damage. Adventitious roots can be induced on plant stems or leaves that were developed from meristematic tissue cells (Pizarro & Díaz-Sala, 2019). Adventitious roots can be regarded as a plant response to environmental stress, infection by pathogenic bacteria, or tissue damage (Steffens & Rasmussen, 2016).

In tissue culture, adventitious roots can be induced by plant hormones applied to the culture medium (Yu et al., 2017). Moreover, the development of adventitious roots can also be regulated by endogenous plant hormones in vivo. Notably, ethylene is a simple gaseous plant hormone that influences many physiological processes, including plant growth, development and senescence (Iqbal et al., 2013). It also influences seed germination (Ribeiro et al., 2018), seedling growth (Gniazdowska et al., 2010), sex expression (especially female flower formation in cucumber) (Pawełkowicz et al., 2019), fruit ripening (Mou et al., 2016), secondary cell wall formation (Felten et al., 2018), the growth of pollen tubes (Jia et al., 2018), root cortical senescence (Schneider et al., 2018), and the rate of photosynthesis (Khan et al., 2016). Ethylene also plays an essential role in plant response and adaptation to biotic and abiotic stress conditions (Tao et al., 2015), including cadmium stress (Alves et al., 2017), water deficit stress (Gao et al., 2017), waterlogging stress (Luan et al., 2018), UV-B stress (Prudnikova et al., 2016) and magnesium deficiency (Liu et al., 2017). Ethylene also promotes the initiation of lateral root primordia derived from the pericycle as well as the emergence and elongation of lateral roots. Many studies have also reported that ethylene may act as a signaling molecule that stimulates adventitious rooting in the mung bean (Robbins et al., 1985), tomato (Negi et al., 2010), tamarack (Calvo-Polanco, Señorans & Zwiazek, 2012), petunia (Druege et al., 2014), *Arabidopsis thaliana* (Rasmussen et al., 2017), peach (Park et al., 2017) and mango (Li et al., 2017). Results from our laboratory have also shown that a 10 µM ethylene treatment significantly enhances adventitious root development in marigolds (Jin et al., 2017). Ethylene and its precursor, 1-aminocyclopropane-1-carboxylic acid (ACC), are involved in the water uptake capacity of roots, which is regulated by relative air humidity; in fact, ethylene and ACC are related to the protein expression of aquaporin in root cells (Calvo-Polanco et al., 2017).

In recent years, proteomic approaches have provided insight into the complex mechanisms underlying biological processes at the protein level. To date, proteomic approaches have been widely employed to study aluminum stress (Wang et al., 2014), drought (Xie et al., 2016), response to waterlogging stress (Xu et al., 2018), grain development (Ma et al., 2014), kernel tissue development (Zhang et al., 2017) and somatic embryogenesis (Zhu et al., 2018).

Studies on the relative gene expression of ethylene-related genes have not only focused on plant vegetative tissues but also on flower senescence and fruit ripening (Hall et al., 2001; Klee, 2004; Stepanova & Alonso, 2005). For example, previous studies have examined the expression of the ethylene receptor gene in tomato plants, the 1-aminocyclopropane-1-carboxylate oxidase gene (*ACO*) in pea (*Pisum sativum* L.) and rape (*Brassica rapa*) plants (Lashbrook, Tieman & Klee, 1998; Petruzzelli et al., 2003; Puga-Hermida et al., 2003), and the S-adenosylmethionine synthetase gene (*SAMS*) in the mango (*Mangifera indica* L.) (Li et al., 2017). Among the ethylene-related enzymes encoded by these genes, SAMS catalyzes the reaction for producing S-adenosylmethionine (SAM) from methionine and ATP. SAM is the precursor for ethylene and polyamine biosynthesis (Van de Poel et al., 2013). The protein expression of SAMS may be related to the development of adventitious roots since its protein expression peaks during the formation of root primordia (Brinker et al., 2004). Therefore, the study of ethylene and ethylene synthesis is of great significance to plant rooting and its regulation. However, few studies have thus far examined genes related to ethylene synthesis in the context of adventitious root formation.

In recent years, researchers have reported the relationship between ethylene biosynthesis and plant photosynthesis. For example, the photosynthetic rate of maize leaves treated with ethephon increased significantly (Feng et al., 2003). The absence of the ethylene receptor in ethylene-insensitive tobacco cells led to a significant decrease in the photosynthetic rate (Tholen et al., 2008). Ethylene has been reported to enhance photosynthetic N-use efficiency and promote photosynthesis in mustard cultivars differing in photosynthetic capacity (Iqbal et al., 2013). Therefore, it is necessary to study the mechanism by which ethylene affects cucumber adventitious root formation. In the present study, the effects of ethylene on adventitious root development in cucumber were evaluated at the molecular level using proteomic techniques. In addition, the indices involved in endogenous ethylene synthesis, photosynthesis, carbon assimilation, and energy metabolism were determined to verify the regulatory role of exogenous ethylene in adventitious root development in cucumber explants.

## Materials and Methods

### Plant materials and growth conditions

Plump and healthy cucumber (*Cucumis sativus* L. ‘Xinchun No. 4’) seeds were selected, washed in distilled water, and surface-sterilized in 5% (w/v) sodium hypochlorite for 10 min. The seeds were then germinated on filter paper moistened with distilled water in Petri dishes and maintained at 25 ± 1 °C and 60% relative humidity for 5 day with a 14 h/10 h (day/night) photoperiod. The photosynthetically active radiation was 200 μmol m^−2^ s^−1^ in the growth chamber (Shanghai Yuejin Medical Instruments Co., Ltd., China). On the 5th day after germination, seedlings with fully spread cotyledons and uniform size were selected. The primary roots of the seedlings were removed, and the seedlings were then grown at the same temperature and photoperiod for another 5 day under different treatments. Seedlings without primary roots were considered as explants. Root number and root length per explant were counted and measured.

### Treatments of explants

Experiment 1: after removing the primary roots, explants were transferred to petri dishes in which the top covers had holes, at a density of 10 explants per petri dish. Each replicate contained six petri dishes, and there were three duplicates in each treatment, which gave 18 petri dishes arranged in a complete randomized design in the growth chamber. Different concentrations (0, 0.1, 0.5, 1, 10 and 50 μM) of the ethylene-releasing compound Ethrel (Shanghai Chemical Reagent Co., Ltd., Shanghai, China) were applied. The control group involved six mL of distilled water in each petri dish. In this experiment, the root number and root length were maximized by treatment with 0.5 μM Ethrel; therefore, 0.5 μM Ethrel was used in the subsequent experiments.

Experiment 2: six treatments were used: (i) the optimum concentration of Ethrel (0.5 μM), which was selected based on the results of Experiment 1; (ii) 1 μM aminoethoxyvinylglycine (AVG) (Merck, Darmstadt, Germany; a competitive inhibitor of 1-aminocyclopropane-1-carboxylic acid synthase (ACS) alone; (iii) 0.1 µM AgNO_3_ (Merck, Darmstadt, Germany; an inhibitor of ethylene) alone; (iv) 0.5 µM Ethrel + 1 µM AVG; (v) 0.5 µM Ethrel + 0.1 µM AgNO_3_; (vi) control (distilled water). The concentrations of AVG (synthetic inhibitor of ethylene) and AgNO_3_ (functional inhibitor of ethylene) were based on previous studies conducted in our lab. Each treatment was replicated three times, with six petri dishes per replicate, in a complete randomized design in the growth chamber. The beginning of the experiment (i.e., time point 0 h) was when the conditions in the growth chamber stabilized (temperature 25 ± 1 °C, relative humidity 60%). Samples of the explants for index determination were taken at 0, 12, 24 and 48 h.

### iTRAQ-based proteome determination and data analysis

The isobaric tags for absolute and relative quantification technique, iTRAQ, is a quantitative analysis of the proteome, which is more accurate and easier to use than the two-dimensional electrophoresis technique (Noirel et al., 2011). According to previous results obtained by our lab, the rooting process of adventitious roots is divided into three phases following excision of the main root: 0–12 h is the root induction phase for adventitious roots; 12–24 h is the root formation phase for adventitious roots; 24–48 h is the root elongation phase. Therefore, the detection of protein expression was conducted in accordance with these developmental phases and performed at the 12, 24 and 48 h time points. After treatment with ethylene, whole seedlings were mixed and collected to extract total proteins. The protein extraction and quantitative analysis samples contained two biological replicates. Each cucumber seedling sample (0.2 g) was fully ground into powder in liquid nitrogen, then 10% TCA (containing 0.07% β-ME) was added to extract total proteins. After extraction, the concentration of each protein sample was measured using a Bicinchoninic acid (BCA) Protein Assay Kit (Bio-Rad, Hercules, CA, USA). Then, 1D SDS-PAGE electrophoresis was used to detect protein degradation in the sample. Proteins (100 μg) were separated on 12.5% SDS-PAGE gel (constant current 14 mA, 90 min). Protein bands were visualized by Coomassie Blue R-250 staining in order to detect protein degradation in the sample. Then, a 100 μg peptide mixture from each sample was labeled using 8-plex iTRAQ reagent according to the manufacturer’s instructions (Applied Biosystems, Framingham, MA, USA). The two biological replicates for the cucumber seedling peptides were labeled as Control 12 h—113, Control 24 h—114, Control 48 h—115; Ethrel 12 h—116, Ethrel 24 h—117, Ethrel 48 h—118. iTRAQ-labeled peptides were fractionated by SCX chromatography using the AKTA Purifier system (GE Healthcare, Chicago, IL, USA). For high-performance liquid chromatography, each fraction was injected for nanoscale liquid chromatography coupled to tandem mass spectrometry (nanoLC-MS/MS) analysis. The peptide mixture was loaded onto a reverse phase trap column (Thermo Scientific Acclaim PepMap100, 100 μm × 2 cm, nanoViper C18) connected to a C18-reversed phase analytical column (Thermo Scientific Easy Column, 10 cm long, 75 μm inner diameter, 3 μm resin) in buffer A (0.1% formic acid) and separated with a linear gradient of buffer B (84% acetonitrile and 0.1% formic acid) at a flow rate of 300 nL/min controlled by IntelliFlow technology. LC-MS/MS analysis was performed on a Q Exactive mass spectrometer (Thermo Scientific, Waltham, MA, USA) coupled to an Easy nLC (Proxeon Biosystems, now Thermo Fisher Scientific) for 120 min. The mass spectrometer was operated in the positive ion mode. According to the protein abundance levels, when the protein difference multiple is ≥1.2 or ≤0.83, the *p*-value is less than 0.05, and significant differences in protein expression between treatments should be determined using further statistical tests (Yang et al., 2013; Chen et al., 2017a; Wang et al., 2019). Bioinformatics analysis results were inquired from UniProt, NCBInr and SwissProt. Mass spectrometry proteomics data have been deposited to the ProteomeXchange Consortium via the PRIDE (Perez-Riverol et al., 2018) partner repository with the dataset identifier PXD016444.

### Real-time qPCR analysis

For q-PCR analysis, we collected three biological replicates per treatment by pooling material collected from six cucumber explants. The cucumber *actin* gene was used as the internal reference gene. The corresponding primers for the genes encoding the candidate proteins, the genes encoding ethylene synthesis-related enzymes, and the genes encoding Calvin cycle-associated enzymes are shown in Table 1. Total RNA extraction and gene expression analysis were performed according to Hu’s method (Hu et al., 2017). The reverse transcription reaction system consisted of 2 µL total RNA, 1 µL 10 mM oligo (dT)_18_, 4 µL 5×RT Reaction MIX, 0.8 µL TUREscript H-Rtase, and 12.2 µL RNase free ddH_2_O. The qPCR experiment was executed according to the manufacturer’s instructions for the fluorescence ration qPCR instrument (LightCycler^®^ 96 System, Roche, UK). The reaction system consisted of 10 µL 2×Tli RNaseH Plus, 0.8 µL forward primer, 0.8 µL reverse primer, 2 µL cDNA, and 6.4 µL RNase Free dH_2_O. The PCR procedure was executed with 3 technical replicates for each biological replicate. The PCR procedures were as follows: initial denaturation at 95 °C for 30 s, then the cycle steps were repeated 40 times (95 °C for 5 s, 60 °C for 30 s), and the melting curve conditions were 95 °C for 5 s, 60 °C for 60 s and 95 °C. The last step of cooling was 50 °C for 30 s. Quantification analysis was performed by the comparative CT method (Livak & Schmittgen, 2001).

**Table 1:** Primer sequences of qPCR analysis in the research.

Gene name	Gene ID	Protein name	Primer pairs
*Csa_6G499090*	101204542	Mitochondrial dicarboxylate carrier protein	F: 5′- GGGCCAATGGCACTTTACAA-3′ R: 5′- TGTTCCAGCGTCACAAACAG-3′
*nad5*	11123919	NADH-ubiquinone oxidoreductase chain 5	F: 5′- TTGCTTGCGGCATCTCTAAC-3′ R: 5′- CCGCATATCTTGCTCATCCG-3′
*Csa_7G419610*	101214617	S-adenosylmethionine synthase	F: 5′- CCTTGTACCGTTGAGCTTCG-3′ R: 5′- ATCGGCAGCGTAGATCTGAA-3′
*Csa_3G099680*	101207278	Chlorophyll a-b binding protein	F: 5′- CAAGCCATTGTGACCGGAAA-3′ R: 5′- TTGGTGGCATAAACCCAAGC-3′
*atpI*	*3429379*	ATP synthase subunit a	F: 5′- TTGCTCACGTCTCGAATGAA-3 R: 5′- GGAAGTCGGCCAACATTTGTA-3′
*Csa_5G457770*	101222001	Aspartokinase	F: 5′- TCAGGTCCTGCATCCACAAT-3′ R: 5′- CTCTGTTCCGGGTGATGAGA-3′
*Csa_6G517340*	105434554	DNA-directed RNA polymerase subunit beta	F: 5′- CACACTTCCTCCGTTACCCT3′ R: 5′- CCCAAGCATCTTCCTGTGTG-3′
*Csa_3G873780*	101213650	Zinc finger protein	F: 5′- GATGACGGTTATGGCGATGG-3′ R: 5′- CAGCAGACCATGTCGAAGAC-3′
*Csa_2G248700*	101208904	Glucose-1-phosphate adenylyltransferase	F: 5′- AGCAACTGCATCAACAGTGG-3′ R: 5′- GCTGCAAGAACCTCCACAAA-3′
*Csa_2G258680*	101211191	40S ribosomal protein S12	F: 5′- CGGTTCGATCGGTTTCTACG-3′ R: 5′- TGTCCATTGGCTCACCAAGA-3′
*Csa_7G343850*	101221055	Pectinesterase	F: 5′- TTGCCTTCTTCTTCCCTGGT-3′ R: 5′- AGCTGAGCATTCTTCTCCGA-3′
*Csa_7G253750*	101214097	Glycosyltransferase	F: 5′- TCCAAGATTGGGACTGCCAT -3′ R: 5′- TCACGGAACGAGAATCACGA -3′
*Csa_5G630860*	101209617	Folylpolyglutamate synthase	F: 5′- TGTCTCCGTTGAAGCCAAAC-3′ R: 5′- GTAGCTGTTGGACGCTACAC-3′
*Csa_6G510320*	101214692	Hexosyltransferase	F: 5′-GAGCCCGTTGCGATTGTTTA-3′ R: 5′-ACGCTCTATGACACCTTGGA-3′
*Csa_1G542510*	101213646	Phloem protein	F: 5′- GGGAATTCAAGGTCGACAAACA-3′ R: 5′-TAGTGGGAGTGGGAGTGAG-3′
*Lec26*	101213646	26 kDa phloem lectin	F: 5′-GGGAATTCAAGGTCGACAAACA-3′ R: 5′-TAGTGGGAGTGGGAGTGAGA-3′
*Csa_1G710160*	105436367	Phloem filament protein	F: 5′-AGCAGCAAACGACAAAGGAG-3′ R: 5′-CAAACTTTGCCACGTCTTGC-3′
*Csa_2G172500*	101207168	Methionine aminopeptidase	F: 5′-CAAATGAGGGCTGCTTGTCA-3′ R: 5′-CGTAGCCAAGAGGTGAAGGA-3′
*Csa_5G633260*	101223124	Glycosyltransferase	F: 5′-GACCAACGAATCCGCTTCAA-3′ R: 5′- CTCATCCTCCGACTGACCTC -3′
*Csa_6G426880*	101220980	Serine/threonine-protein phosphatas	F: 5′- ATTGAGCGGATGGGAGAGAG-3′ R: 5′- AAAGCTGCTAGAGGAAGCCA-3′
*Csa_2G421020*	101213629	Peroxidase	F: 5′- CTGAGAGGGATTCTGCACCA-3′ R: 5′-ATCTCTTCGGCCAGTTGGAA -3′
*ClCa*	101210008	Chloride channel protein	F: 5′-GATTCCCTGTTGTGGATGCC-3′ R: 5′- AGTACTAGTCCGTGCAGCTC-3′
*Csa_3G166240*	101211228	DNA-directed RNA polymerase subunit	F: AAGCTCTTGTGTTCGGCTTG-3′ R: GCTAGGGCTTTAGGAGCTGA-3′
*ACO1*	101221653	1-Aminocyclopropane-1-carboxylate oxidase	F: 5′- AGCCAGCAAAGGATTGAACG-3′ R: 5′- CGATGTTGGAGACTGGGAGA-3′
*ACS2*	101217331	ACC synthase	F: 5′- TTCTCCTCCGACGAGTTCAC-3′ R: 5′- CGGTGGTGACGACTTTATCG-3′
*ETR1*	101213479	Ethylene receptor	F: 5′- GTGCTAGACAATGGCGTGTT-3′ R: 5′- CTCTGCTTCTCTTCGGGCTA-3′
*ERS*	101205786	Ethylene response sensor	F: 5′- GCTGTTGCACTTTCACATGC-3′ R: 5′- CTCTGCTTCTCTTCGGGCTA-3′
*rbcS*	101219300	Ribulose-1, 5-bisphosphate carboxylase/oxygenase small subunit	F: 5′- GCCTCTCAGACTCAACACCA-3′ R: 5′- CGGAAGATTTGAGGCCAGTG-3′
*GAPDH*	101202856	Glyceraldehyde-3-phosphate dehydrogenase	F: 5′- GAAGCACATTGAGGCTGGAG-3′ R: 5′- GACTCGTCATGGCTGTATGC-3′
*FBA*	101219476	Fructose-1,6-bisphosphate aldolase	F: 5′- AAGCGACTGGCAAGCATAAG-3′ R: 5′- TTGAAGCACTTCCACGAACG-3′
*TK*	101215805	Transketolase	F: 5′- ATGCAATGGGATTGCCCTTC-3′ R: 5′- TGGGTTGGACCATCTTCTCC-3′
*actin*	DQ641117	Actin	F: 5′-CCCATCTATGAGGGTTACGCC-3′ R: 5′-TGAGAGCATCAGTAAGGTCACGA-3′

### Western blot analysis

The same samples used in the iTRAQ analysis were used for the western blotting analyses. Two differentially expressed proteins (DEPs), SAMS (A0A0A0K5X0) and AtpA (A0A0A0L6I8), were selected. The TCA/acetone method was used to extract total proteins. The protein concentrations were measured using a BCA Protein Assay Kit (Beyotime Biotechnology, China). Under alkaline conditions, divalent copper ions (Cu^2+^) can be reduced to monovalent copper ions (Cu^+^) by protein; then, a chelation reaction occurs between Cu^+^ and BCA, which produces a purple compound, for which the maximum absorbance is proportional to the protein concentration (Smith et al., 1985). The extracted total proteins were separated by SDS-PAGE and then transferred onto a polyvinylidene difluoride (PVDF) membrane. Membranes were then incubated overnight at 4 °C with polyclonal antibodies at the appropriate dilution against S-adenosylmethionine synthase (SAMS; 1:5,000) and ATP synthase subunit a, chloroplastic (AtpA; 1:5,000) (Agrisera, Uppsala, Sweden). The PVDF membrane was rinsed with 1×TBST (0.1%). This was then incubated with goat anti-rabbit IgG (H&L) and horseradish peroxidase (HRP)-conjugated secondary antibody diluted 1:3,000 for 1 h. The color was developed using an electrochemical luminescence (ECL) kit. Finally, the developed films were scanned (BioRad, Hercules, CA, USA) and precisely quantified using the PDQUEST 6.0 software package (BioRad, Hercules, CA, USA).

### Ethylene content

Ethylene content was determined as described by Bu et al. (2013) with some modifications. Cucumber explants (0.5 g) were placed in penicillin bottles for 2 h at 25 ± 1 °C. Then, one mL gas was extracted using a syringe from the top of the bottle for detection in a gas chromatograph (Agilent Technologies Inc., Santa Clara, CA, USA).

### Activities of ethylene synthesis related enzymes

The activity of ACC synthase (ACS) was measured according to the method described by Tian et al. (2004) with some modifications. Fresh samples (0.5 g) were ground in liquid nitrogen with 100 mM PBS. The mixture was centrifuged for 20 min at 2,500*×g* at 4 °C. The reaction system consisted of one mL of the supernatant, 1.5 mL reaction liquid (including 250 μM S-adenosylmethionine and 10 μM pyridoxine phosphate), which was then sealed tightly in a penicillin bottle (10 mL) and kept in a water bath at 30 °C for 1 h. After that, 0.1 mL of 25 mM HgCl_2_ was added and kept in an ice bath for 10 min. Then, 0.4 mL of another solution (5% NaOCl:saturated NaOH = 2:1, v/v) was added to the mixture and kept in an ice bath for 3 min. Finally, one mL of gas was extracted to determine the amount of ethylene produced.

ACC oxidase (ACO) was determined using the method described Zhang, Yuan & Leng (2009) with some modifications. Fresh cucumber explant samples (0.5 g) were ground with three mL of 0.1 M Tris-HCl (pH 7.2) in an ice bath. The mixture was centrifuged for 20 min at 12,000 rpm. The supernatant (0.5 mL) was mixed with 1.5 mL reaction liquid (consisting of 10% glycerol, 30 mM ascorbic acid, 30 mM NaHCO_3_, 2 mM ACC and 0.1 mM FeSO_4_) in a penicillin bottle (10 mL) and kept in a water bath at 30 °C for 0.5 h. Then, one mL of gas was extracted to determine the amount of ethylene produced.

### Chlorophyll content

A 0.2 g fresh sample was shredded and soaked in 15 mL of 80% acetone in a test tube. The test tube was sealed and kept in the dark. When the sample turned white, 80% acetone was added to a final volume of 25 mL. The absorbance of the supernatant was measured at 646 and 663 nm. The formulas for calculating the chlorophyll a and chlorophyll b content are as follows (Lichtenthaler & Wellburn, 1985):

Chlorophyll a (mg g FW^−1^) = (12.21 × A_663_ − 2.81 × A_646_) × V/FW

Chlorophyll b (mg g FW^−1^) = (20.13 × A_646_ − 5.03 × A_663_) × V/FW

where V (L) is the extraction volume, and FW (g) is the fresh weight of the sample.

### Chlorophyll fluorescence parameters

Chlorophyll fluorescence parameters were determined according to the method described by Wu et al. (2018) with some modifications. The explants were kept in darkness for 30 min to fully open the photosystem II reaction center before measurement. An Imaging-PAM Chlorophyll Fluorometer (Walz, Effeltrich, Germany) was used to detect the chlorophyll fluorescence parameters. The saturation pulse was 2,700 μM m^−2^ s^−1^. After the saturation pulse, indices such as F0 (minimum fluorescence of the dark-adapted leaves) and Fm (maximum fluorescence yield of the dark-adapted leaves) were obtained. Then, the explants were kept under actinic light (56 μM m^−2^ s^−1^) for 5 min, which was turned on for 0.8 s every 20 s. After this light-adaptation process, indices including F0′ (minimum fluorescence of the light-adapted leaves), Fs (steady chlorophyll fluorescence of light-adapted leaves) and Fm′ (maximum fluorescence yield of the light-adapted leaves) were collected. The maximum photochemical efficiency of PSII (Fv/Fm), effective quantum yield of PSII (ФPSII), and photochemical quenching (qP) were calculated according to the following formulas (Hu et al., 2017):

Fv/Fm = (Fm − F0) / Fm

ФPSII = (Fm′ − Fs) / Fm′

qP = (Fm′ − Fs) / (Fm′ − F0′)

### Activities of Calvin cycle-related enzymes

Enzyme extraction was performed according to the method described by Rao & Terry (1989) with some modifications. A fresh sample (0.5 g) of cucumber explants was ground in liquid nitrogen. Then, five mL of the 4 °C precooled solution was added to extract the enzyme (0.4 mM EDTA-Na_2,_ 100 mM Hepes-Na buffer, 10 mM MgCl_2_, 1% PVP, 0.1% BSA, 100 mM Na-ascorbate, 50 mM DTT). Then, the mixture was centrifuged for 5 min at 12,000*×g* at 4 °C, and the supernatant was collected for determination of enzyme activity. Following ELISA tests, the activities of ribulose bisphosphate carboxylase oxygenase (Rubisco), glyceraldehyde-3-phosphate dehydrogenase (GAPDH), fructose-1, 6-diphosphate (FBPase), fructose-1,6-bisphosphate aldolase (FBA) and transketolase (TK) were measured using determination kits (Shanghai Yanji Biological Technology Ltd., China).

### Soluble sugar, starch and soluble protein content

The content of soluble sugar and starch was measured according to the methods described Van Handel (1968) and Paul, Driscoll & Lawlor (1991) with few modifications. Each fresh cucumber sample (0.2 g) was shredded and kept in a boiling water bath with 10 mL distilled water for 15 min. Then, the mixture was centrifuged for 5 min at 3,000 rpm min^−1^. The supernatants and precipitates from the three centrifugations were collected for soluble sugar and starch determinations, respectively. The supernatant was evaporated at 80 °C to reduce the volume to 20 mL. We withdrew one mL of the solution, transferred it to a test tube, and then added 1.5 mL distilled water, 0.5 mL anthrone ethyl acetate and five mL concentrated sulfuric acid. The test tube was fully oscillated, and the soluble sugar content was determined after cooling. The absorbance was measured at 630 nm, and the soluble sugar content was calculated according to the standard curve.

The precipitate from the previous step was collected for starch determination. Distilled water (three mL) was added to the precipitate. After keeping the resuspended precipitate in a boiling water bath for 15 min, two mL of 9.2 M perchloric acid was added to extract starch for 15 min. Then, we added 10 mL distilled water to the mixture, centrifuged for 10 min at 3,000 rpm min^−1^ and collected the supernatant. The supernatant was filtered, and the volume was adjusted to 25 mL in a volumetric bottle. The reaction system conditions and determination methods were the same as those used in the measurement of soluble sugars. Finally, the starch content was calculated according to the standard curve.

Soluble protein content was measured according to the method described by Bradford (1976) with some modifications. A fresh sample (0.2 g) of cucumber explants was ground in liquid nitrogen, and six mL of distilled water was then added. The homogenate was centrifuged for 10 min at 4,000 rpm min^−1^. The supernatant was collected and set to 10 mL. The solution (0.1 mL) was thoroughly mixed with 0.9 mL distilled water and five mL Coomassie brilliant blue. Then, the absorbance was measured at 595 nm, and the soluble protein content was calculated according to the standard curve.

### Statistical analysis

The results were expressed as mean values (±SE). Except for the determination of mRNA expression levels of DEPs, we tested for significant differences between treatment means using the variance of multivariate variables with the LSD method. To examine the changes in gene and protein expression from qPCR and iTRAQ, respectively, over time, we applied Tukey’s test to determine significant differences in the means (*P* value < 0.05) between time points to validate that the proteomic DEP data concurred with the mRNA expression level data generated by qPCR. SPSS 22.0 was employed for data analysis, and all figures were created using OriginPro 2017 (OriginLab Institute Inc., Northampton, MA, USA).

## Results

### Adventitious root development

**Figure 1:**
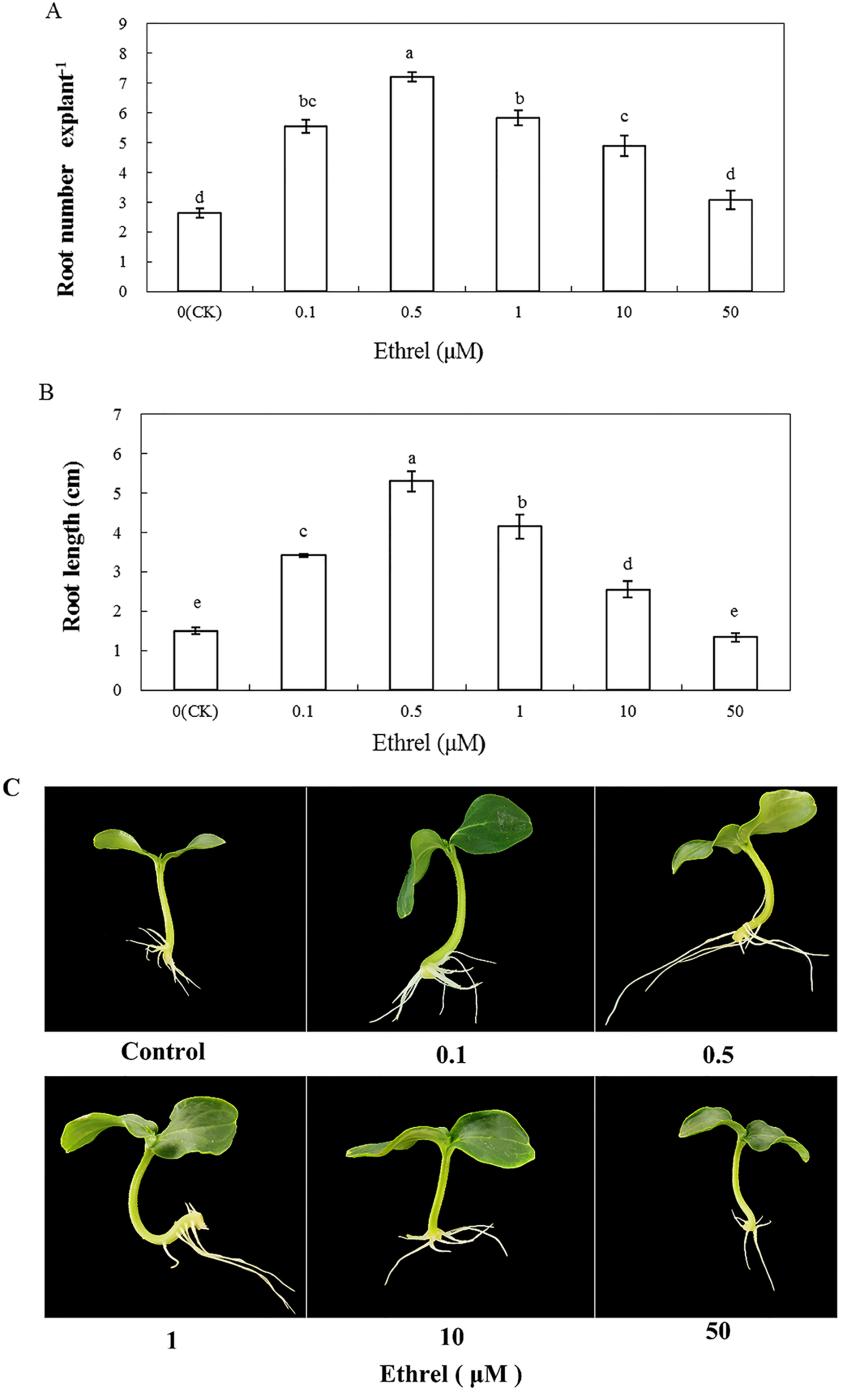
Effects of different concentrations of ethrel on adventitious root development in cucumber explants. The primary root system was removed from hypocotyls of 5-day-old germinated cucumber. Explants were incubated with distilled water or different concentrations of ethrel for 5 day. (A) Adventitious root number and (B) adventitious root length. Data were expressed as mean ± SE (*n* = 3, each replication revealed mean of 10 explants). Bars indicate the SE. Significant differences (*P* < 0.05) between treatments are indicated by different lowercase letters. Photographs (C) were taken after 5 day of treatment.

We applied the ethylene-releasing compound Ethrel at concentrations of 0, 0.1, 0.5, 1, 10 and 50 μM to cucumber explants. The cucumber explants treated with 0.1, 0.5, 1 and 10 μM Ethrel produced more and longer adventitious roots than the control explants (Fig. 1). However, there were no significant differences in either root number or root length between the control and the 50 μM Ethrel-treated explants. Among the different concentrations, the maximum root number and root length were both observed at 0.5 μM Ethrel, under which they were increased by 176.9% and 253.3%, respectively, compared with the control. Subsequently, 0.5 μM Ethrel was used for further experiments.

**Figure 2:**
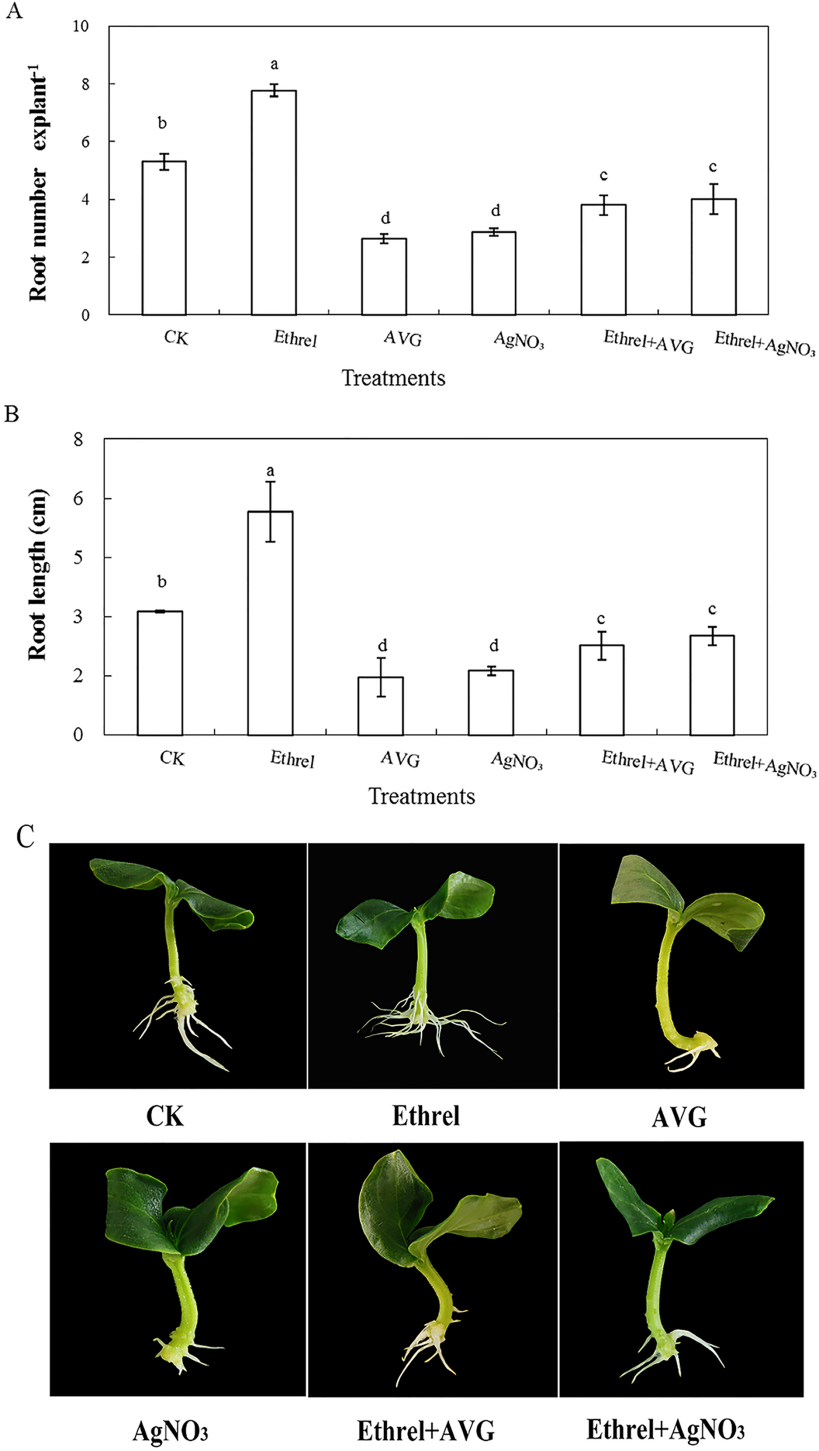
Effects of ethrel, AVG and AgNO_3_ on adventitious root development in cucumber explants. The primary root system was removed from hypocotyls of 5-day-old germinated cucumber. Explants of cucumber were incubated with distilled water, 0.5 µM Ethrel, 1 µM AVG and 0.1 µM AgNO_3_ for 5 day. (A) Adventitious root number and (B) adventitious root length. Photographs (C) were taken after 5 day of treatment. Data were expressed as mean ± SE (*n* = 3, each replication revealed mean of 10 explants). Bars indicate the SE. Significant differences (*P* < 0.05) between treatments are indicated by different lowercase letters.

In order to investigate the effect of ethylene on adventitious rooting in cucumber, an ethylene synthesis inhibitor (AVG) and an action inhibitor (AgNO_3_) were applied in the second experiment (Fig. 2). Compared with the control, AVG and AgNO_3_ significantly inhibited the development of adventitious roots. The root numbers in AVG- and AgNO_3_-treated explants were 50.4% and 45.8% lower than those of the control. Compared with the control, the root lengths of explants treated with AVG and AgNO_3_ decreased by 53.5% and 48.4%, respectively. Furthermore, AVG and AgNO_3_ suppressed the promotive effects of exogenous ethylene and resulted in a significant reduction in adventitious root number and length. These findings further indicated that the adventitious rooting process was positively regulated by ethylene.

### Protein identification and functional annotation

**Figure 3:**
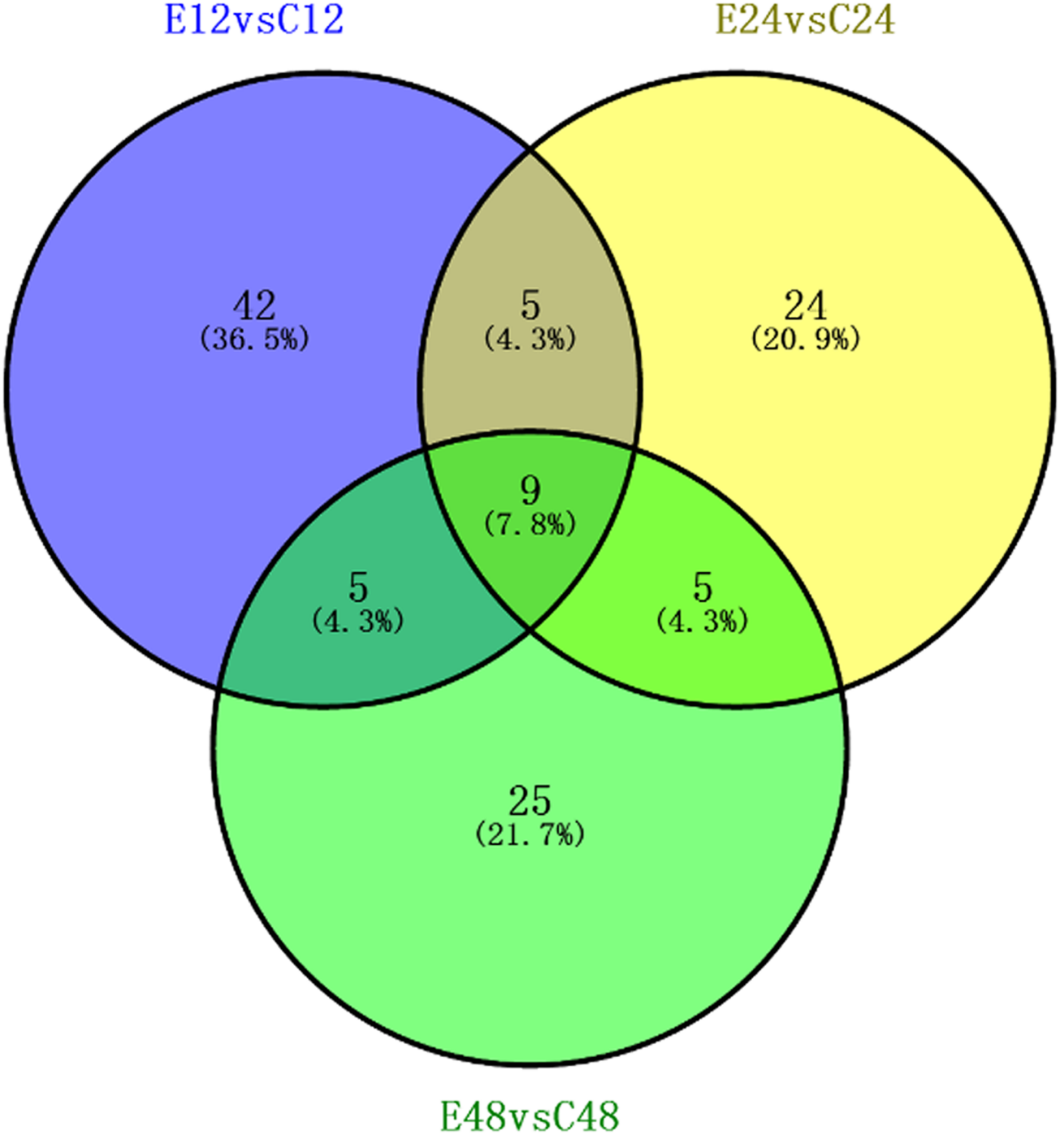
Association analysis of the differential expression of known protein during adventitious rooting phases in cucumber explant treated by ethylene. E12: the root induction phase (RIP) of ethylene treatment; C12: the root induction phase of control. E24: the root formation phase (RFP) of ethylene treatment; C24: the root formation phase of control; E48: the root elongation phase (REP) of ethylene treatment; C24: the root elongation phase of control.

**Figure 4:**
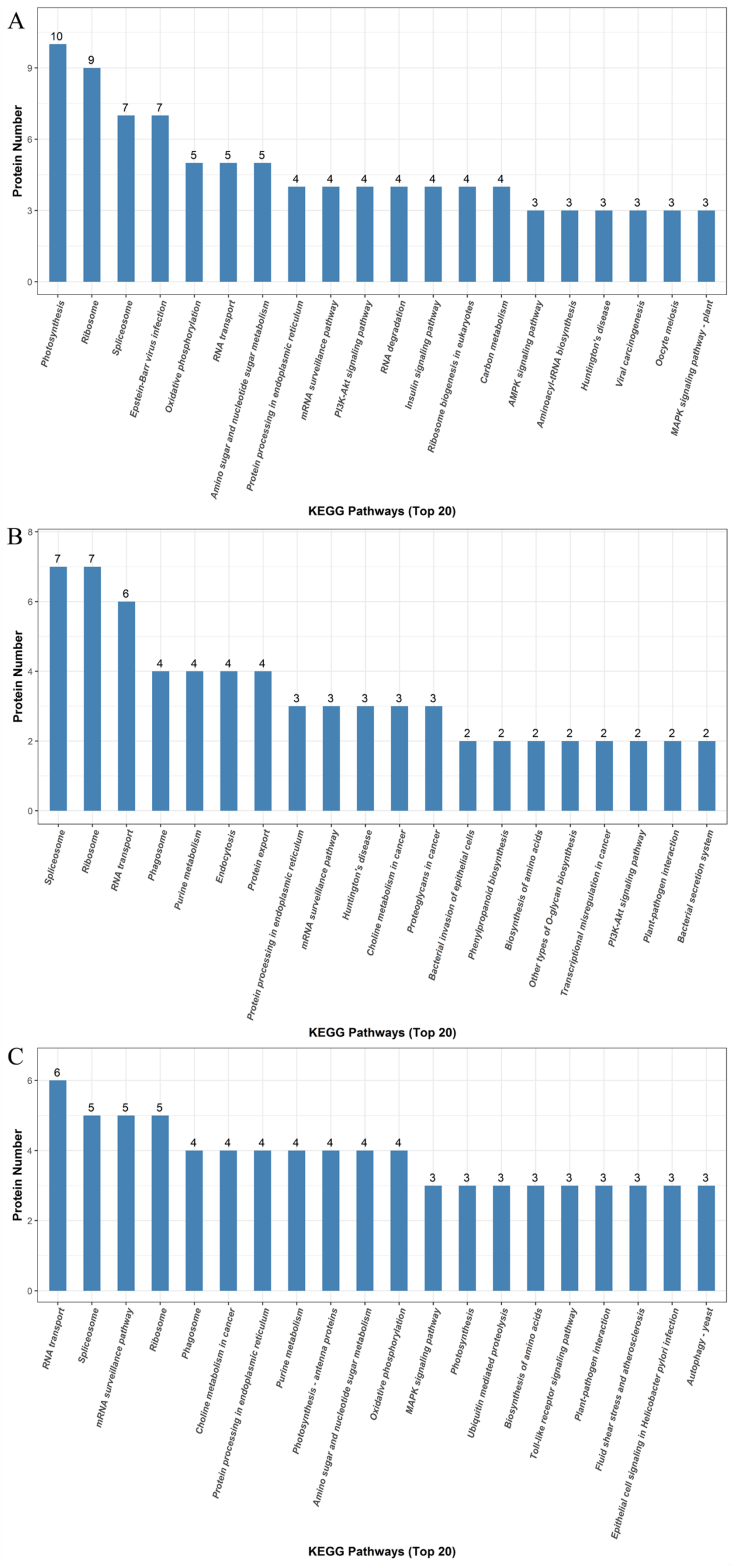
KEGG pathway enrichment analysis of the DEPs detectedin cucumber explants. The protein groups are categorized based on their putative functions. (A) Differentially expressed proteins at the RIP. (B) Differential expression proteins at the RFP. (C) Differentially expressed proteins at the REP.

We identified a total of 5,014 proteins using the iTRAQ technique. Proteins with abundances that had changed by more than 1.2-fold or less than 0.83-fold with significant *P*-values (<0.05) were selected. Based on these criteria, 821 DEPs were selected, and they were differentially abundant at different time points during rooting. To identify the selected DEPs, we consulted the Uniprot database to obtain accession numbers and protein names for these DEPs; these are listed in Table S1. The DEP comparison groups between treatments were sorted based on the time point (i.e., 12, 24, or 48 h post treatment), so that we compared the differences between the ethylene treatment and the control at 12h (E12 vs. C12), 24 h (E24 vs. C24) and 48 h (E48 vs. C48). There were 115 known DEPs, and their changes in abundance are listed in Table 2. In the E12 vs. C12 comparison, 61 DEPs were identified, out of which 37 proteins (61.0%) were upregulated and 24 proteins (39.3%) were downregulated; in the E24 vs. C24 comparison, 43 DEPs were identified, out of which 16 proteins (37.2%) were upregulated and 27 proteins (63.0%) were downregulated, whereas 44 DEPs were identified in the E48 vs. C48 comparison, out of which 25 proteins (56.8%) were upregulated and 19 proteins (43.2%) were downregulated. Figure 3 illustrates the intersections of the detected DEPs among the three comparison groups. There were nine proteins commonly regulated in the three comparable groups. Figure 4 shows the KEGG functional classifications of the DEPs in different rooting periods. Moreover, functional analysis of the DEPs was carried out using biological process, molecular function, and cellular component according to the GO database in different rooting periods were applied in the Supplemental Files. The main KEGG functional classifications of the DEPs at 12 h were photosynthesis, ribosome and spliceosome (Fig. 4A); at 24 h were spliceosome, ribosome and RNA transport (Fig. 4B); at 48 h were RNA transport, spliceosome and mRNA surveillance pathway (Fig. 4C). Therefore, we focused on the photosynthesis of seedling in further study. And in order to explore the effects of ethylene on adventitious rooting, we also focused on the ethylene biosynthesis and role receptors.

**Table 2:** Differentially expressed proteins and corresponding genes during the induction of adventitious roots of cucumber explants affected by ethylene.

Accession	Description	Gene	E12 vs. C12	E24 vs. C24	E48 vs. C48
A0A0A0KIS8	Mitochondrial dicarboxylate carrier protein	*Csa_6G499090*	↑	↓	↑
G3EIX1	NADH-ubiquinone oxidoreductase chain 5	*nad5*	↑	↑	↓
A0A0A0K5X0	S-adenosylmethionine synthase	*Csa_7G419610*	↑	↑	↓
A0A0A0L6I8	Chlorophyll a-b binding protein, chloroplastic	*Csa_3G099680*	↑	↑	↓
Q4VZP5	ATP synthase subunit a, chloroplastic	*atpI*	↑	↑	↓
A0A0A0LVH4	Cystatin Hv-CPI6	*Cystatin Hv-CPI6*	↓	↑	↓
A0A0A0KTT0	Aspartokinase	*Csa_5G457770*	↓	↓	↑
A0A0A0KNB9	DNA-directed RNA polymerase subunit beta DNA	*Csa_6G517340*	↓	↓	↑
A0A0A0LGJ0	Zinc finger protein	*Csa_3G873780*	↓	↓	↑
A0A0A0LJ10	Glucose-1-phosphate adenylyltransferase	*Csa_2G248700*	↑	↑	–
A0A0A0LM61	40S ribosomal protein S12	*Csa_2G258680*	↑	↓	–
A0A0A0K6S6	Pectinesterase	*Csa_7G343850*	↑	↓	–
A0A0A0K3P3	Glycosyltransferase	*Csa_7G253750*	↓	↑	–
A0A0A0KTX7	Folylpolyglutamate synthase	*Csa_5G630860*	↓	↓	–
A0A0A0KJQ8	Hexosyltransferase	*Csa_6G510320*	↑	–	↑
A0A0A0LVN2	Phloem protein	*Csa_1G542510*	↑	–	↓
Q8LK68	26 kDa phloem lectin (Fragment) 2	*Lec26*	↑	–	↓
A0A0A0LYF4	Phloem filament protein	*Csa_1G710160*	↑	–	↓
A0A0A0LI62	Methionine aminopeptidase	*Csa_2G172500*	↓	–	↓
A0A0A0KS62	Glycosyltransferase	*Csa_5G633260*	–	↑	↑
A0A0A0KEW9	Serine/threonine-protein phosphatase	*Csa_6G426880*	–	↓	↑
A0A0A0LMY0	Peroxidase	*Csa_2G421020*	–	↓	↑
I1Z8C8	Chloride channel protein	*ClCa*	–	↓	↑
A0A0A0L816	DNA-directed RNA polymerase subunit	*Csa_3G166240*	–	↓	↑
Q8S3W3	Phenylalanine ammonia lyase 1 (Fragment)	*PAL1*	↑	–	–
A0A0A0KHV5	Pectinesterase)	*Csa_6G514890*	↑	–	–
Q4VZJ2	30S ribosomal protein S12, chloroplastic)	*rps12-A*	↑	–	–
A0A0A0LPD9	Cysteine protease	*Csa_2G363570*	↑	–	–
A0A0A0KA08	40S ribosomal protein S12	*Csa_7G432030*	↑	–	–
A0A0A0KYQ6	Carbon catabolite repressor protein	*Csa_4G508480*	↑	–	–
A0A0A0KP40	Peptidyl-prolyl cis-trans isomerase	*Csa_5G202380*	↑	–	–
A0A0A0M324	DnaJ	*Csa_1G642540*	↑	–	–
A0A0A0LD22	Histone H2A	*Csa_3G408540*	↑	–	–
A0A0A0LW64	40S ribosomal protein S25	*Csa_1G573590*	↑	–	–
A0A0A0K7B3	Phloem filament protein	*Csa_7G222870*	↑	–	–
Q6UNT3	Hypersensitive-induced response protein	*Csa_6G404210*	↑	–	–
A0A0A0KBP8	Poly(A)-binding protein C-terminal interacting protein 6	*Csa_6G013350*	↑	–	–
A0A0A0L9J7	60S ribosomal protein L6	*Csa_3G698560*	↑	–	–
P00293	Plastocyanin	*PETE*	↑	–	–
V5RFY5	Plasma intrinsic protein 1-2	*PIP1-2*	↑	–	–
A0A0A0KP38	Global transcription factor group	*Csa_6G525380*	↑	–	–
A0A0A0LAX1	Major latex protein	*Csa_3G319290*	↑	–	–
A0A0A0KZA2	Potassium transporter	*Csa_4G107490*	↑	–	–
Q9SLQ8	Oxygen-evolving enhancer protein 2, chloroplastic	*PSBP*	↑	–	–
A0A0A0KI44	Mitochondrial pyruvate carrier	*Csa_6G489900*	↑	–	–
A0A0A0LWS0	GTP-binding nuclear protein	*Csa_1G304710*	↑	–	–
A0A0A0K565	Chlorophyll a-b binding protein, chloroplastic	*Csa_7G033300*	↑	–	–
A0A0A0LYU4	GRIP and coiled-coil domain-containing protein	*Csa_1G560750*	↑	–	–
A0A0A0KKA0	AMP dependent CoA ligase	*Csa_5G154230*	↑	–	–
Q4VZH7	Photosystem II reaction center protein L	*psbL*	↓	–	–
A0A0A0KU52	Protein CLP1 homolog	*Csa_5G635390*	↓	–	–
A0A0A0KWC7	Glutamate dehydrogenase	*Csa_4G025140*	↓	–	–
Q4VZJ0	Protein PsbN	*psbN*	↓	–	–
A8JP99	Plasma membrane ATPase	*HA3*	↓	–	–
A0A0A0L7Q1	ATP-dependent (S)-NAD(P)H-hydrate dehydratase)	*Csa_3G149940*	↓	–	–
A0A0A0KP21	Oleosin	*Csa_5G523090*	↓	–	–
A0A0A0L5X9	4-hydroxy-4-methyl-2-oxoglutarate aldolase	*Csa_3G044520*	↓	–	–
A0A0A0KL78	ATP synthase gamma chain ATP	*Csa_6G513760*	↓	–	–
A0A0A0LC78	ATP-dependent Clp protease proteolytic subunit	*Csa_3G653410*	↓	–	–
A0A0A0KC20	Cysteine proteinase inhibitor	*Csa_6G022340*	↓	–	–
A0A0A0K4K4	Acyl-[acyl-carrier-protein] hydrolase	*Csa_7G343320*	↓	–	–
A0A0A0KQF0	Chloroplast small heat shock protein class I	*Csa_5G198120*	↓	–	–
B0F832	Eukaryotic initiation factor iso4E	*eIF(iso)4E*	↓	–	–
Q4VZH4	Photosystem I assembly protein Ycf3	*ycf3*	↓	–	–
A0A0A0KC19	Cytochrome P450	*Csa6G088170*	↓	–	–
A0A0A0LJY3	Eukaryotic translation initiation factor 6	*EIF6*	↓	–	–
Q4VZH6	Cytochrome b559 subunit beta	*psbF*	–	↑	–
Q96398	Chromoplast-specific carotenoid-associated protein, chromoplastic	*CHRC*	–	↑	–
A0A0A0K5Z0	Carboxypeptidase	*Csa_7G420830*	–	↑	–
A0A0A0KU38	Ribosomal protein L15	*Csa_3G112770*	–	↑	–
A0A0A0LQP6	Cytochrome P450	*Csa1G044890*	–	↑	–
P42051	Photosystem I reaction center subunit psaK, chloroplastic (Fragment)	*PSAK*	–	↑	–
A0A0A0KCU9	Basic blue protein	*Csa_6G344240*	–	↑	–
A0A0A0KUY0	Protein translocase subunit SecA	*Csa_4G050230*	–	↑	–
A0A0A0L6Z3	Flavin-containing monooxygenase	*Csa_3G033780*	–	↓	
A0A0A0LRI5	Inositol-tetrakisphosphate 1-kinase	*Csa_2G439740*	–	↓	–
A0A0A0L368	Chalcone-flavonone isomerase family protein	*Csa_4G622760*	–	↓	–
A0A0A0L048	Cytokinin riboside 5’-monophosphate phosphoribohydrolase	*Csa_4G646190*	–	↓	–
A0A0A0LHZ5	Protein kinase	*Csa_2G005900*	–	↓	–
Q4VZN5	30S ribosomal protein S14, chloroplastic	*rps14*	–	↓	–
A0A0A0KYP0	Phloem lectin	*Csa_4G501830*	–	↓	–
A0A0A0LGI2	Glycosyltransferase	*Csa_3G855310*	–	↓	–
A0A0A0K3Z5	Peroxidase	*Csa_7G061710*	–	↓	–
A0A0A0LK87	Diacylglycerol kinase	*Csa_2G058110*	–	↓	–
A0A0A0L546	Thiamine thiazole synthase, chloroplastic	*THI1*	–	↓	–
A0A0A0L2I9	Tubulin beta chain	*Csa_4G307390*	–	↓	–
A0A0A0LKQ2	Nucleolar GTP-binding protein 1	*Csa_2G351000*	–	↓	–
A0A0A0KJB6	Histone H1	*Csa_6G505280*	–	↓	–
A0A0A0LHP0	RuvB-like helicase	*Csa_3G824760*	–	↓	–
A0A0A0KGX7	Purple acid phosphatase	*Csa_6G366500*	–	↓	–
A0A0A0KWB2	Oleosin	*Csa_4G023040*	–	–	↑
A0A0A0K515	U1 small nuclear ribonucleoprotein C	*Csa_7G390140*	–	–	↑
A0A0A0L4C8	Peptidyl-prolyl cis-trans isomerase	*Csa_3G125010*	–	–	↑
A0A0A0L4U6	Ferredoxin-1	*Csa_3G146700*	–	–	↑
A0MCW3	Pathogen induced 4 protein	*pi4*	–	–	↑
A0A0A0L505	MFP1 attachment factor 1	*Csa_4G641730*	–	–	↑
A0A0A0L7A7	PRA1 family protein PRA1	*Csa_3G143550*	–	–	↑
A0A0A0KA91	Cytochrome P450	*Csa6G088710*	–	–	↑
A0A0A0LPY4	Nascent polypeptide-associated complex subunit beta	*Csa_1G031860*	–	–	↑
A0A0A0KGJ2	DNA ligase	*Csa_6G148180*	–	–	↑
A0A0A0KZG2	Pectate lyase	*Csa_4G293240*	–	–	↑
Q40559	Peroxidase	*pre-peroxidase*	–	–	↑
A0A0A0LJG3	Rac-type small GTP-binding protein	*Csa_2G270750*	–	–	↑
A0A0A0KDT1	Autophagy-related protein 3	*Csa_6G104635*	–	–	↑
A0A0A0LCK0	DNA helicase	*Csa_3G722880*	–	–	↑
A0A0A0L0U5	Chlorophyll a-b binding protein, chloroplastic	*Csa_4G664570*	–	–	↓
A0A0A0LS25	Tonoplast intrinsic protein	*Csa_2G374630*	–	–	↓
A0A0A0LX69	V-type proton ATPase subunit F	*Csa_1G611810*	–	–	↓
A0A0A0K773	Phloem protein 2	*Csa_7G056500*	–	–	↓
A0A0A0K762	Glycosyltransferase	*Csa_7G051410*	–	–	↓
A0A0A0KG08	Diacylglycerol kinase	*Csa_6G123470*	–	–	↓
A0A0A0KV98	Chlorophyll a-b binding protein, chloroplastic	*Csa_5G589390*	–	–	↓
A0A0A0K3W6	Phloem filament protein	*Csa_7G222880*	–	–	↓
A0A0A0KKC3	Threonine dehydratase	*Csa_6G448740*	–	–	↓
A0A0A0KKP5	Ribosomal protein L15	*Csa_5G162630*	–	–	↓

**Note:**

The comparison groups of DEPs: ethylene treatment compared with CK at 12h (E12 vs. C12), at 24 h (E24 vs. C24) and at 48 h (E48 vs. C48). In this table, “↑” indicates increased proteins, and “↓” indicates significant decreased protein (fold change ≥ 1.20 or ≤ 0.83 and *p* value < 0.05). “−” indicates the difference expression was not significant.

### Validation of the DEP data with mRNA expression data

**Figure 5:**
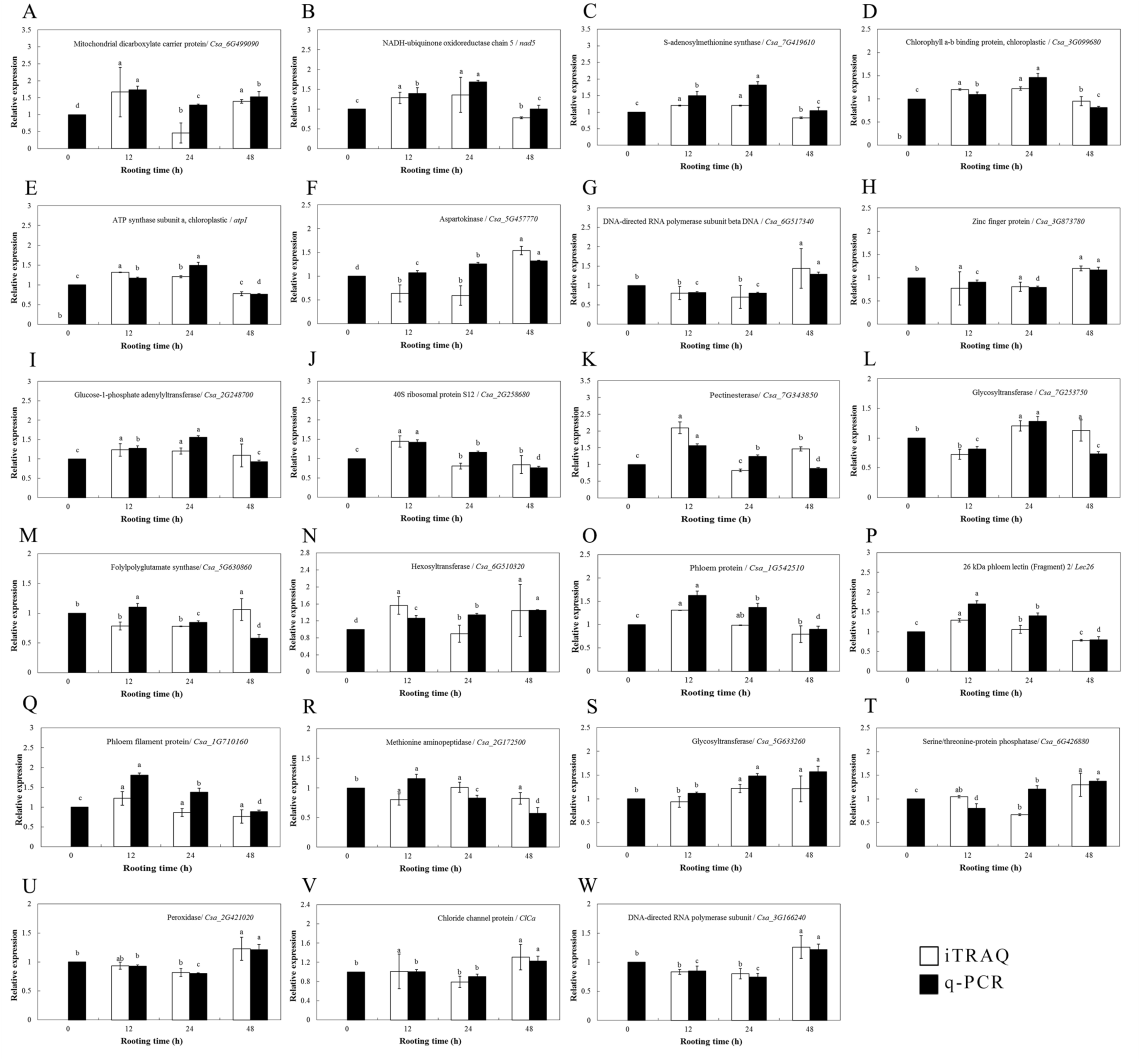
Protein expression levels of interesting proteins and their corresponding genes expression levels during adventitious root development in cucumber explants. (A) Mitochondrial dicarboxylate carrier protein/ Csa_6G499090. (B) NADH-ubiquinone oxidoreductase chain 5/ nad5. (C) S-adenosylmethionine synthase/ Csa_7G419610. (D) Chlorophyll a-b binding protein, chloroplastic/ Csa_3G099680. (E) ATP synthase subunit a, chloroplastic/ atpI. (F) Aspartokinase/ Csa_5G457770. (G) DNA-directed RNA polymerase subunit beta DNA/ Csa_6G517340. (H) Zinc finger protein/ Csa_3G873780. (I) Glucose-1-phosphate adenylyltransferase/ Csa_2G248700. (J) 40S ribosomal protein S12/ Csa_2G258680. (K) Pectinesterase/ Csa_7G343850. (L) Glycosyltrasferase/ Csa_7G253750. (M) Folypolyglutamate synthase/ Csa_5G630860. (N) Hexosyltransferase/ Csa_6G510320. (O) Phloem protein/ Csa_1G542510. (P) 26 kDa phloem lectin (Fragment) 2/ Lec 26. (Q) Phloem filament protein/ Csa_1G710160. (R) Methionine aminopeptidase/ Csa_2G172500. (S) Glycosyltranferase/ Csa_5G633260. (T) Serine/ threonine-protein phosphatase/ Csa_6G426880. (U) Peroxidase/ Csa_2G421020. (V) Chloride channel protein/ ClCa. (W) DNA-directed RNA polymerase subunit/ Csa_3G166240. Data were expressed as mean ± SE (n = 3). Bars indicate the SE. Significant differences (*P* < 0.05) between time points are indicated by different lowercase letters.

The transcriptional levels of the 24 selected genes were surveyed by qPCR and were compared to the expression of DEPs to validate the DEP data (Fig. 5). Among them, 23 genes encoding these proteins were cloned successfully according to the NCBI database, and their expression patterns during ethylene-induced adventitious rooting were investigated at the mRNA level using qPCR. Only one protein (cystatin Hv-CPI6; identified as protein folding, modification, degradation and location-related proteins) was not successfully cloned. These genes involved nine functional categories. Among them, the transcriptional levels of 19 genes showed the same trends as those for the expression of their proteins; these included the proteins involved in carbohydrate and energy metabolism, including NADH-ubiquinone oxidoreductase chain 5 (*nad5*), glucose-1-phosphate adenylyltransferase (*Csa_2G248700*), pectinesterase (*Csa_7G343850*), glycosyltransferase (*Csa_7G253750*), hexosyltransferase (*Csa_6G510320*), and glycosyltransferase (*Csa_5G633260*); the proteins involved in amino acid metabolism, including SAMS (*Csa_7G419610*), DNA-directed RNA polymerase subunit beta (*Csa_6G517340*) and DNA-directed RNA polymerase subunit (*Csa_3G166240*); stress defense-related proteins, such as phloem protein (*Csa_1G542510*), 26 kDa phloem lectin (*Lec26*), and phloem filament protein (*Csa_1G710160*); photosynthesis-related proteins such as chlorophyll a-b binding protein, chloroplastic (*Csa_3G099680*) and ATP synthase subunit a, chloroplastic (*atpI*); transcription- and translation-related related proteins, including 40S ribosomal protein S12 (*Csa_2G258680*); stress response proteins, like peroxidase (*Csa_2G421020*); membrane-associated transporter proteins, such as mitochondrial dicarboxylate carrier protein (*Csa_6G499090*); the ion transport/homeostasis proteins, like the chloride channel protein (*ClCa*); and proteins related to protein folding, modification, degradation, and location, such as the zinc finger protein (*Csa_3G873780*). For some proteins, however, the levels of mRNA expression were different from those of their proteins; these included aspartokinase (*Csa_5G44457770*), folylpolyglutamate synthase (*Csa_5G630860*), pectinesterase (*Csa_7G343850*), hexosyltransferase (*Csa_6G510320*), phloem filament protein (*Csa_1G710160*) and serine/threonine-protein phosphatase (*Csa_6G426880*).

### Protein expression of SAMS and endogenous ethylene production

**Figure 6:**
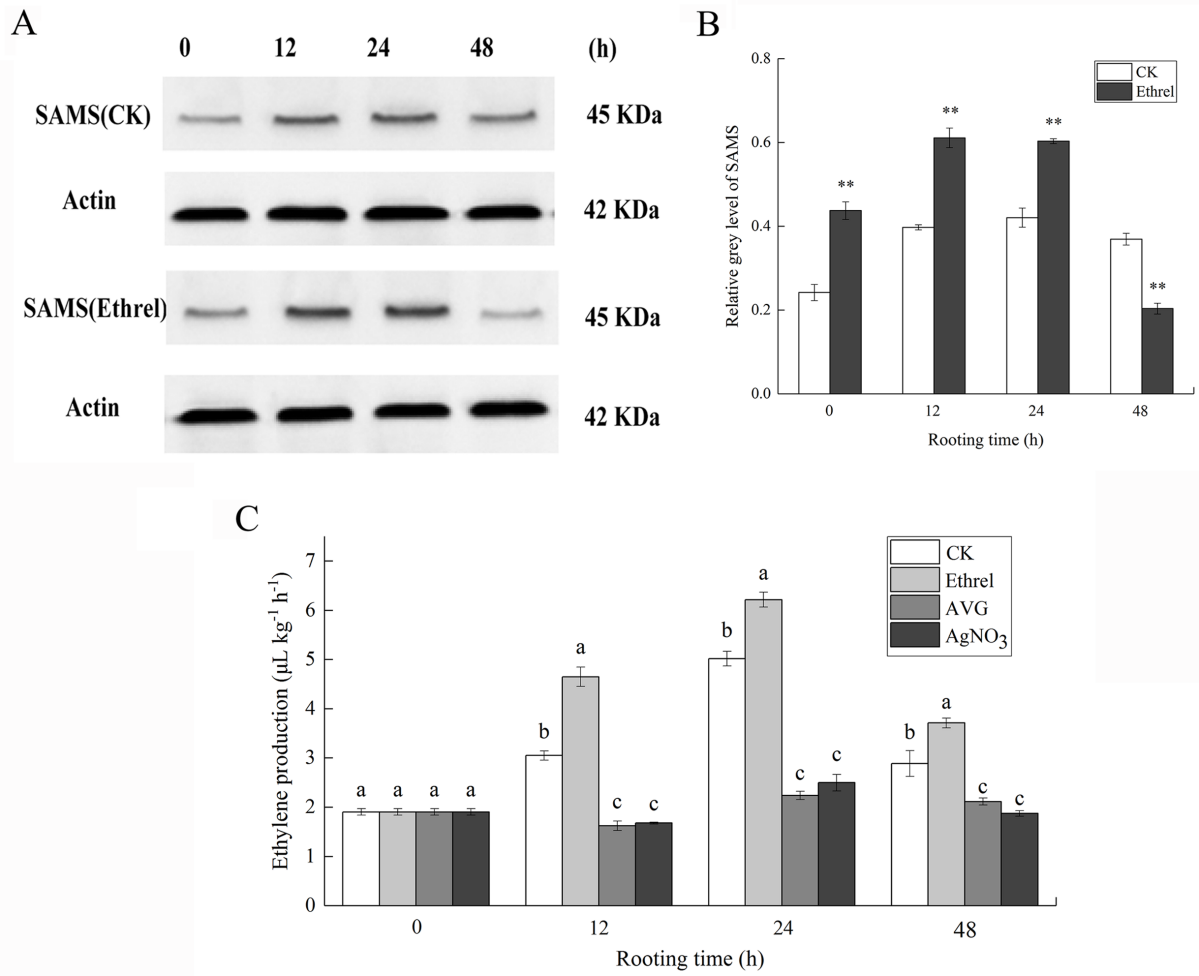
The protein expression of SAMS and ethylene production in cucumber explants. (A) The bands of SAMS protein under different treatments. (B) The histograms showing the ratio of grey level to actin grey level of SAMS protein expression. The value represents the mean ± SE. Significant differences between treatments are indicated by asterisk (*), “*” represents *P* < 0.05, “**” represents *P* < 0.01. (C) The ethylene production in cucumber explants. The value represents the mean ± SE (*n* = 3). Significant differences (*P* < 0.05) between treatments are indicated by different lowercase letters.

S-adenosylmethionine synthase was selected as a candidate protein and its protein expression levels were analyzed by western blotting. Total protein content of the ethylene-treated explants and controls were extracted 0, 12, 24 and 48 h after ethylene treatment. As shown in Fig. 6, the abundance of SAMS increased at 12 and 24 h in explants treated with ethylene, compared with that in the control. In contrast, the abundance of SAMS decreased at 48 h in explants treated with ethylene, relative to that in the control (Figs. 6A and 6B). The western blot results showed that the abundance of SAMS was changed in a manner that was consistent with the iTRAQ results.

As shown in Fig. 6C, in each rooting phase, a sharp increase in ethylene production was observed under the ethrel treatment, and ethylene production reached its highest level at 24 h. For example, ethylene production in the ethrel treatments was 23.9% higher than that of the control at 24 h. Compared with the control, AVG and AgNO_3_ treatments significantly decreased the production of ethylene by 46.9% and 44.9%, respectively, at 12 h. The results indicate that exogenous ethylene application can induce the production of endogenous ethylene.

### Activities and gene expressions of ethylene synthesis related enzymes

**Figure 7:**
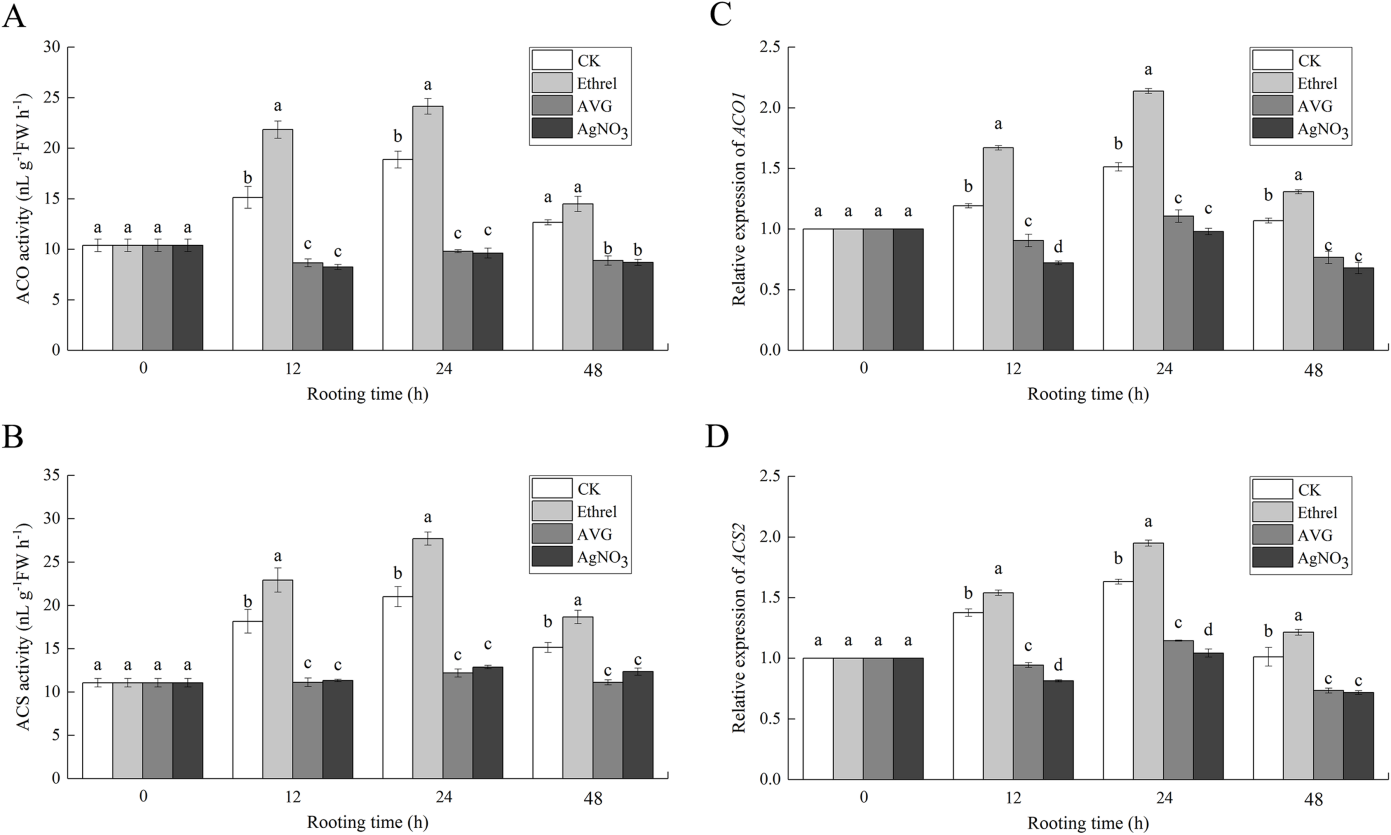
The enzymatic activities of ACO, ACS and their encoding gene expression levels in cucumber explants. (A and B) The enzymatic activities of ACO1 and ACS. (C and D) The relative gene expression levels of *ACO1* and *ACS2*. Each value represents the mean of three independent replicates ± SE (*n* = 3). Bars indicate the SE. Significant differences (*P* < 0.05) between treatments are indicated by different lowercase letters.

Figures 7A and 7B show that the increase in ACO and ACS activities occurred in the control and ethrel treatments at 12 and 24 h but had decreased by 48 h after treatment. The maximum levels of ACO and ACS activities were observed at 24 h after ethrel treatment. However, the time course experiments showed that in AVG and AgNO_3_-treated explants, low levels of ACO and ACS activities were recorded in the cucumber explants. For example, the ACO activities in cucumbers treated with AVG and AgNO_3_ were 48.1% and 49.1% lower than that of the control at 24 h, respectively. Similarly, the ACS activities of the AVG and AgNO_3_ treatments were 41.9% and 38.7% lower than that of the control at 24 h, respectively. These results suggest that AVG and AgNO_3_ could decrease ethylene synthesis by suppressing ACO and ACS activities during adventitious rooting.

To examine the molecular mechanism of ethylene-induced adventitious root development, we analyzed the changes in expression of two ethylene synthesis-related enzymes (*ACO1* and *ACS2*) using qPCR (Figs. 7C and 7D). When ethrel was applied alone, the relative expression of the *ACO1* and *ACS2* genes was significantly higher than that of any other treatments. Compared with the control, the expression levels of *ACO1* and *ACS2* genes increased by 41.3% and 19.5% at 24 h, respectively. However, compared with the control, *ACO1* and *ACS2* expression was significantly decreased by the applications of AVG and AgNO_3_.

### Expression of ethylene receptor-related genes

**Figure 8:**
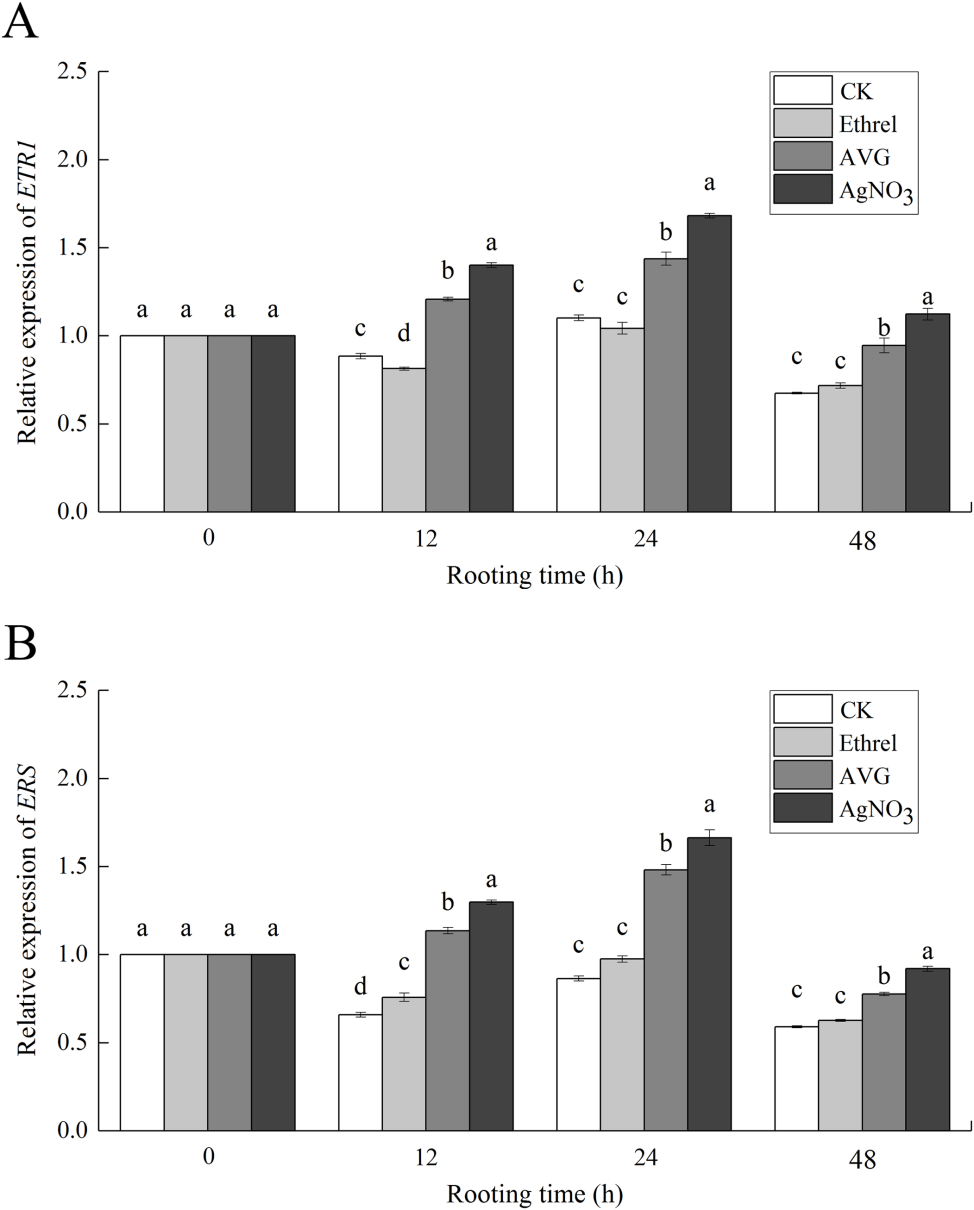
The relative gene expression levels of ethylene receptors *ETR1* and *ERS* in cucumber explants. (A) Relative gene expression level of *ETR1*. (B) Relative gene expression level of *ERS*. Each value represents the mean of three independent replicates ± SE (*n* = 3). Bars indicate the SE. Significant differences (*P* < 0.05) between treatments are indicated by different lowercase letters.

The relative expression levels of *ETR1* and *ERS*, the genes encoding the ethylene receptors ETR1 and ERS, were analyzed to investigate the molecular mechanism of ethylene-induced adventitious rooting (Fig. 8). Compared with the control group, exogenous ethylene inhibited the expression of *ETR1* and *ERS*. Under the ethylene treatment, *ETR1* and *ERS* expression at 12 h had decreased by 17.0% and 12.3%, respectively, compared with their expression at 0 h. However, AVG and AgNO_3_ treatments upregulated their expression. At 24 h, AVG and AgNO_3_ had increased the expression levels of *ETR1* by 27.0% and 33.0%, respectively, and the expression levels of *ERS* by 42.0% and 33.2%, respectively.

### AtpA protein expression

**Figure 9:**
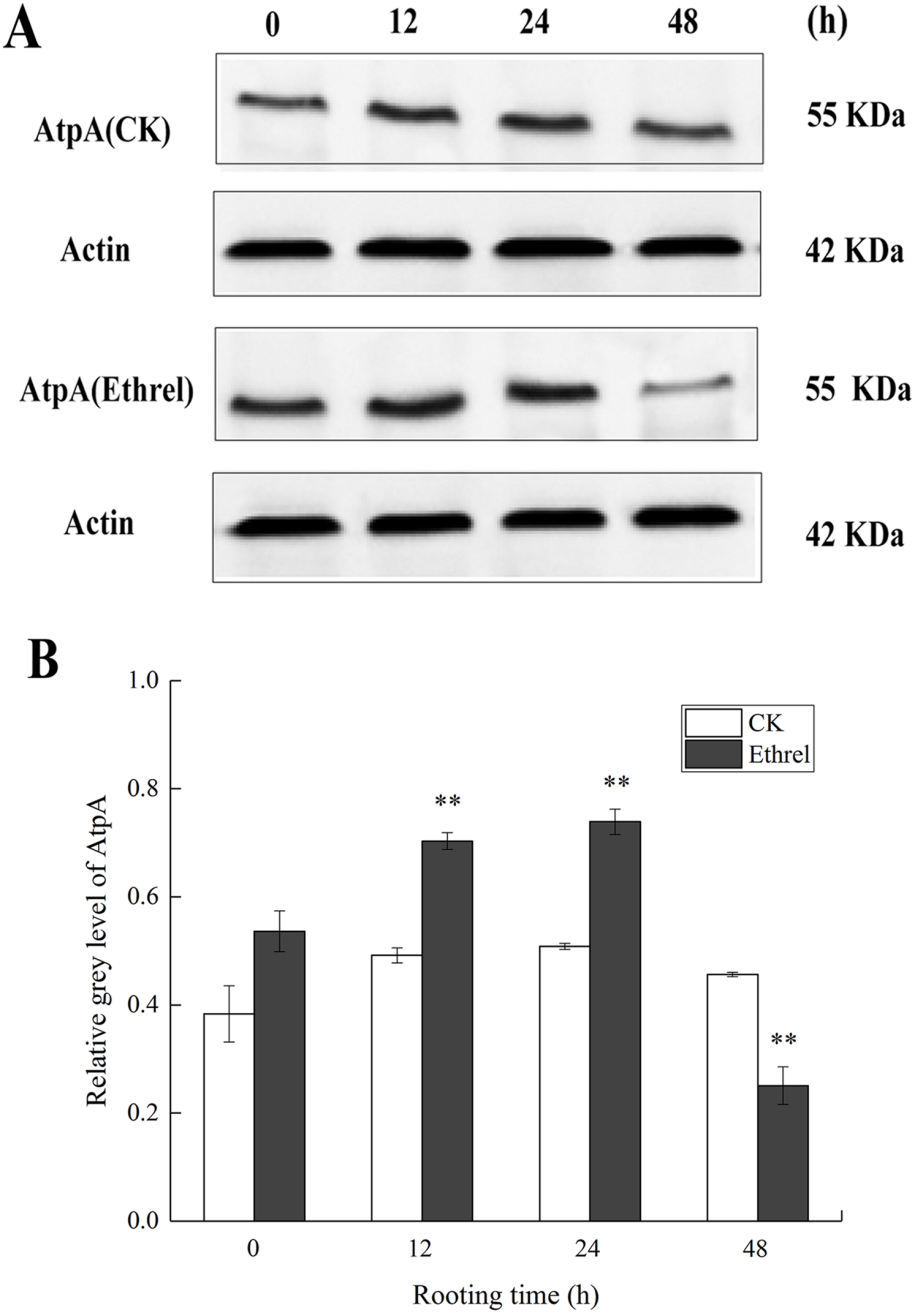
The protein expression level of AtpA in cucumber explants. (A) The bands of AtpA protein under different treatments. (B) The histograms showing the ratio of grey level to actin grey level of AtpA protein expression. The value represents the mean ± SE (*n* = 3). Significant differences between treatments are indicated by asterisk (*), “*” represents *P* < 0.05, “**” represents *P* < 0.01.

We verified the iTRAQ results by assessing the abundance of AtpA through western blotting, using the same protein samples used in the iTRAQ analysis. Compared with the control, the abundance of AtpA was progressively upregulated at 12 and 24 h in the ethylene treatment (Fig. 9). At 48 h, its expression level was downregulated compared to that in the control. In comparison with the results obtained using proteomics, the changes in the trends in the abundance of the AtpA protein were consistent with the observations made by iTRAQ. Thus, the western blot results were in accordance with the iTRAQ results, supporting the reliability of the proteomic data.

### Chlorophyll content and chlorophyll fluorescence parameters

**Figure 10:**
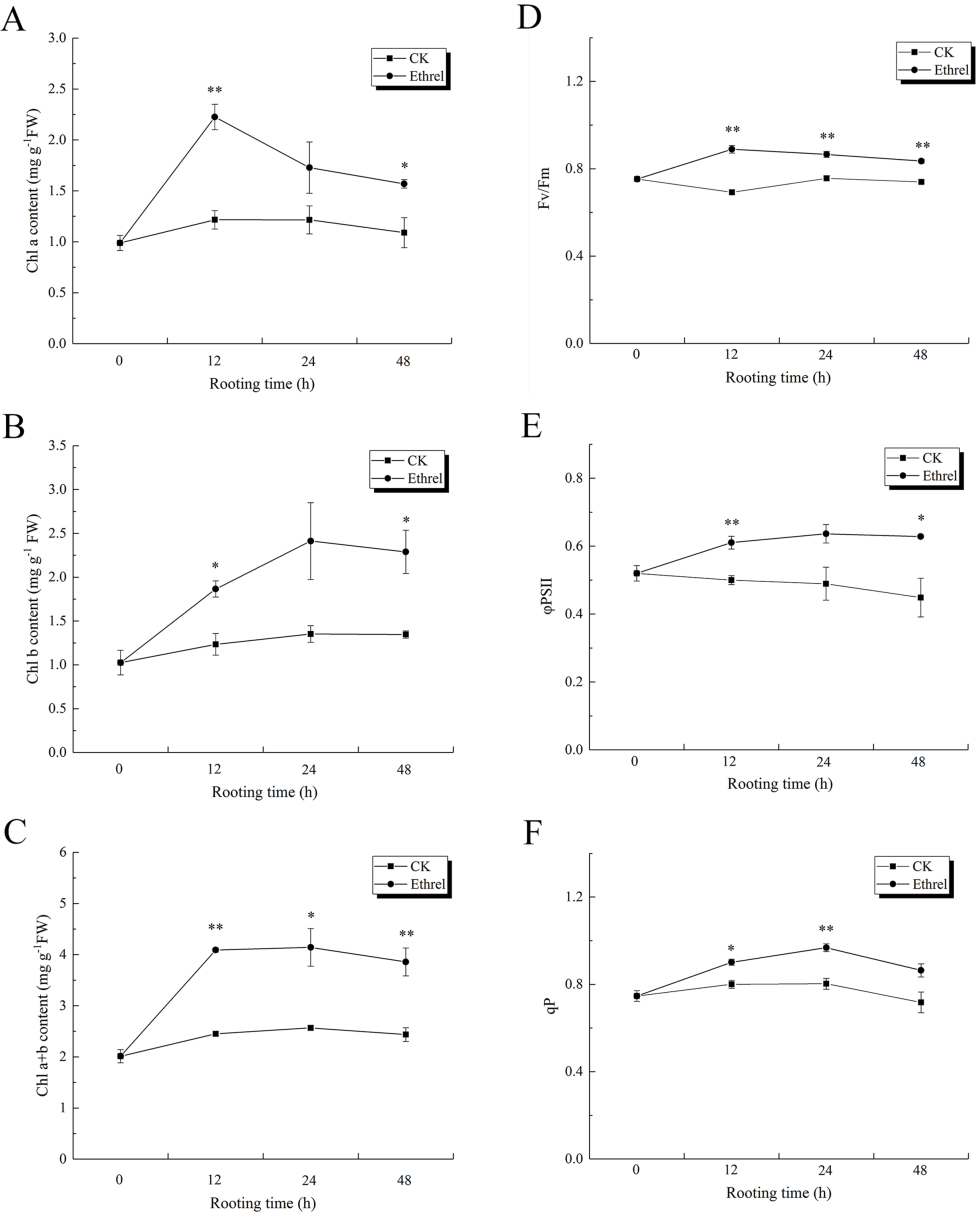
The contents of chlorophyll and flurescence parameters in cucumber explants. (A) The content of chlorophyll a (Chl a). (B) The content of chlorophyll b (Chl b). (C) Total chlorophyll content (Chl a+b). (D) The maximum quantum yield of PSII (Fv/Fm). (E) The effective quantum yield of PSII (φPSII). (F) The photochemical quenching (qP). The value represents the mean ± SE (*n* = 3). Significant differences between treatments are indicated by asterisk (*), “*” represents *P* < 0.05, “**” represents *P* < 0.01.

As shown in Figs. 10A, 10B and 10C, the content of chlorophyll *a*, chlorophyll *b*, and chlorophyll (*a* + *b*) in explants treated with ethylene significantly increased during the 0–48 h period, relative to the control. The maximum level of chlorophyll *a* was observed at 12 h after ethylene treatment, and it rapidly decreased until 48 h. Chlorophyll *b* content increased gradually over the first 24 h but then had declined by the 48 h time point in the ethylene-treated explants. At 24 h, the chlorophyll *b* content of the seedlings treated with ethylene was 78.5% higher than that of the control. The highest value of chlorophyll (*a* + *b*) was observed in the ethylene treatment at 12 h, when it was 66.9% higher than that of the control. Explants treated with ethylene showed significantly higher chlorophyll fluorescence parameter values than the control explants (Figs. 10D–10F). The value of Fv/Fm peaked at 12 h in the ethylene-treated explants, at which time it was 28.9% higher than that of the control. Compared with the control, the value of ΦPSII in the ethylene treatment was elevated by 30.6% at 24 h. In addition, the value of qP in ethylene-treated explants at 24 h was 21.3% higher than that of the control.

### Activities and gene expression levels of Calvin cycle-related enzymes

**Figure 11:**
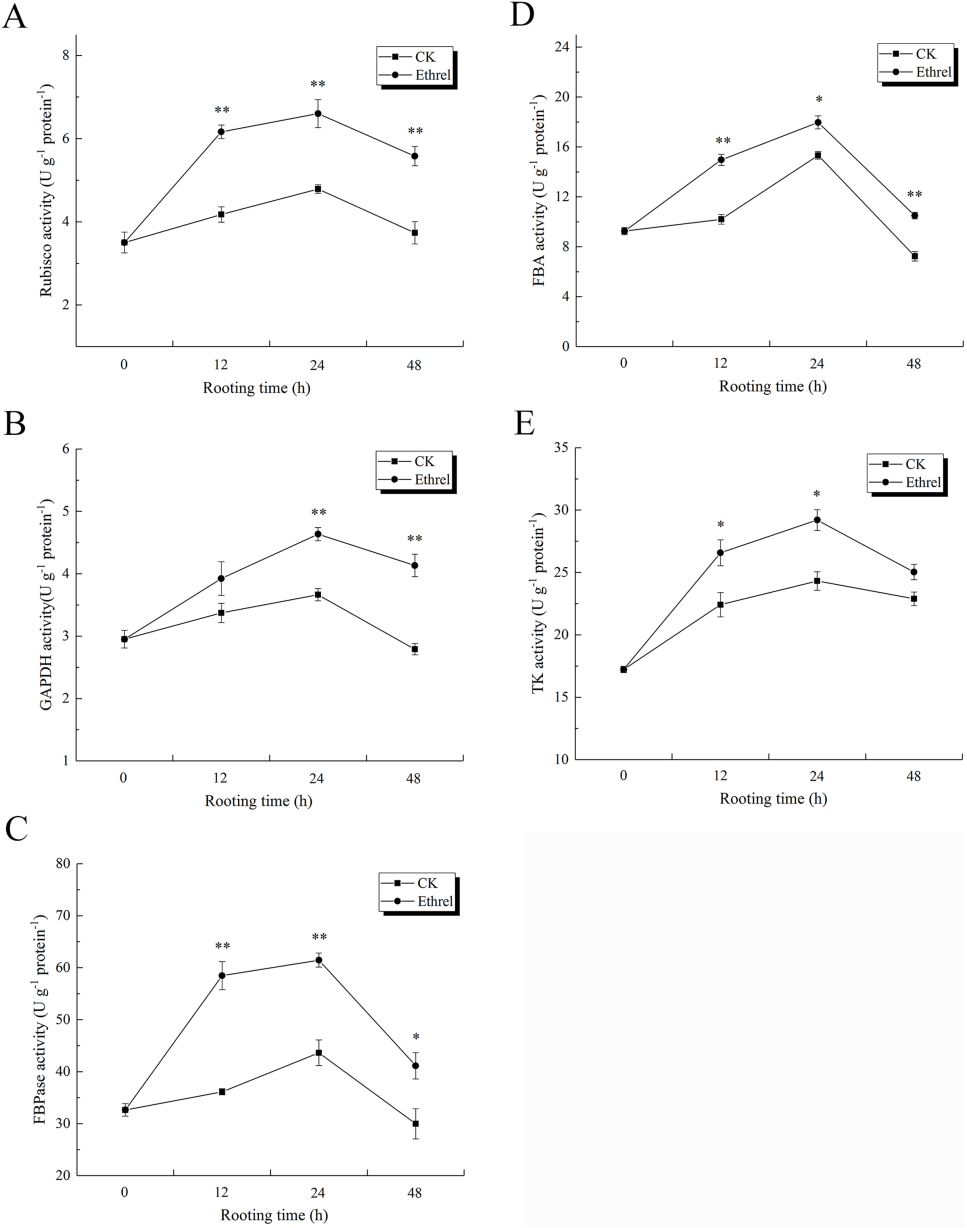
The activities of Calvin cycle related key enzymes in cucumber explants. (A) The activity of Rubisco. (B) The activity of GAPDH. (C) The activity of FBPase. (D) The activity of FBA. (E) The activity of TK. The value represents the mean ± SE (*n* = 3). Significant differences between treatments are indicated by asterisk (*), “*” represents *P* < 0.05, “**” represents *P* < 0.01.

As shown in Fig. 11A, Rubisco activity sharply increased in the ethylene treatment, reaching its highest level at 24 h and then decreasing until 48 h. At 12 h, the ethylene treatment had increased Rubisco activity 47.4% over the level observed in the control. The activity of GAPDH in explants treated with ethylene was significantly higher than that of the control. GAPDH activity under the ethylene treatment was 26.5% higher than that of the control at 24 h (Fig. 11B). In addition, similar to the trend observed for Rubisco activity during the rooting process, the highest level of FBPase activity was observed in the ethylene treatment at 24 h, when it was 40.9% higher than that of the control (Fig. 11C). The ethylene treatment significantly increased FBA activity. At 24 h, the FBA activity of explants treated with ethylene was 17.6% higher than that of the control (Fig. 11D). In addition, TK activity in the ethylene-treated explants was 20.2% higher than that of the control at 24 h.

**Figure 12:**
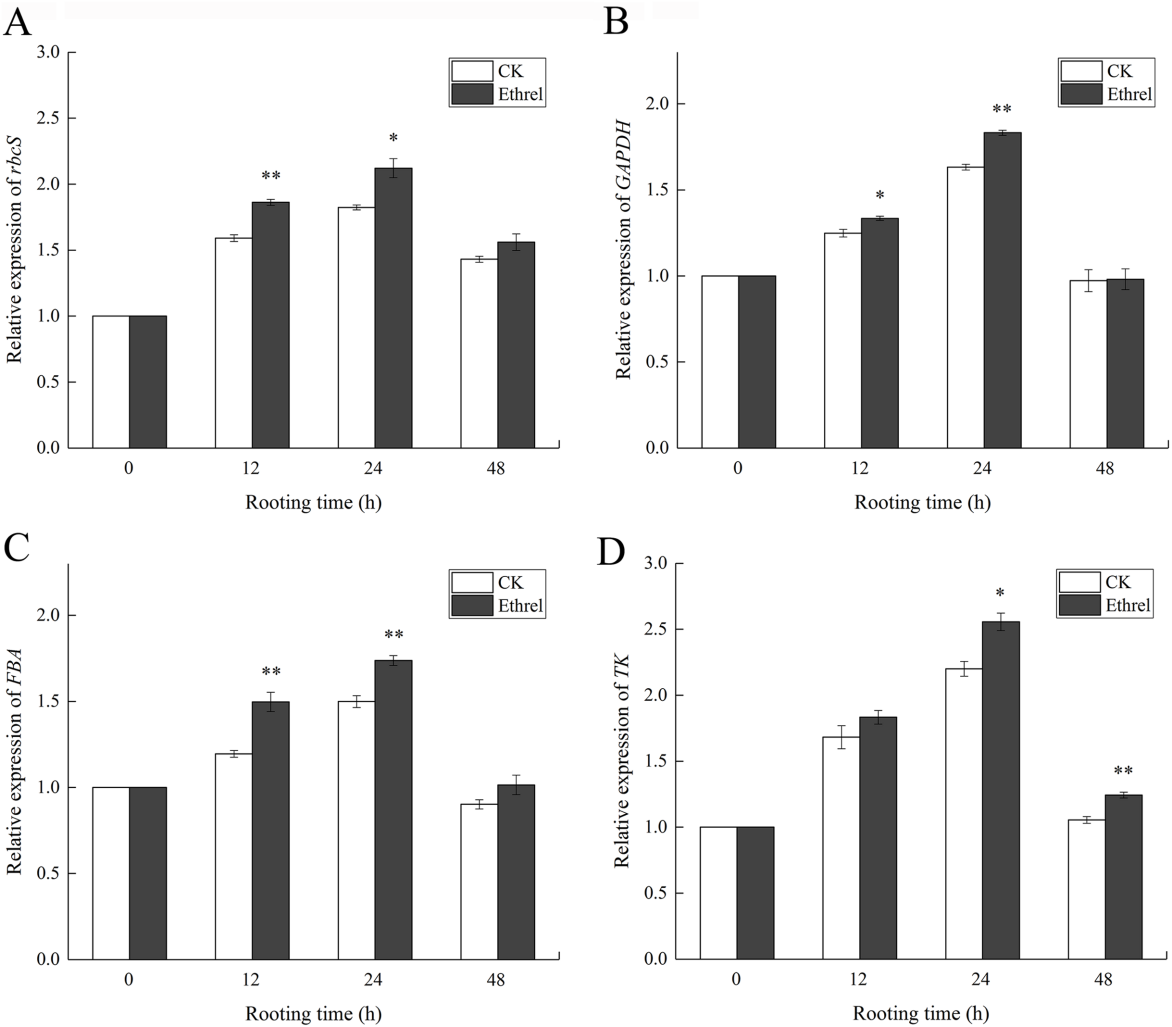
Relative expression levels of genes encoding Calvin cycle related enzymes. (A) The relative expression level of *rbcS*. (B) The relative expression level of *GAPDH*. (C) The relative expression level of *FBA*. (D) The relative expression level of *TK*. The value represents the mean ± SE (*n* = 3). Significant differences between treatments are indicated by asterisk (*), “*” represents *P* < 0.05, “**” represents *P* < 0.01.

The effects of ethylene on the relative expression levels of Calvin cycle-related genes are shown in Fig. 12. The relative expression levels of *rbcS*, *GAPDH*, *FBA*, and *TK* were all upregulated in ethylene-treated explants within 0–24 h and then downregulated within 24–48 h. When compared with the control, the relative expression levels of *rbcS*, *GAPDH*, *FBA* and *TK* in the ethylene treatment increased by 16.3%, 12.2%, 16.0% and 15.4% at 24 h, respectively.

### Content of metabolic constituents

**Figure 13:**
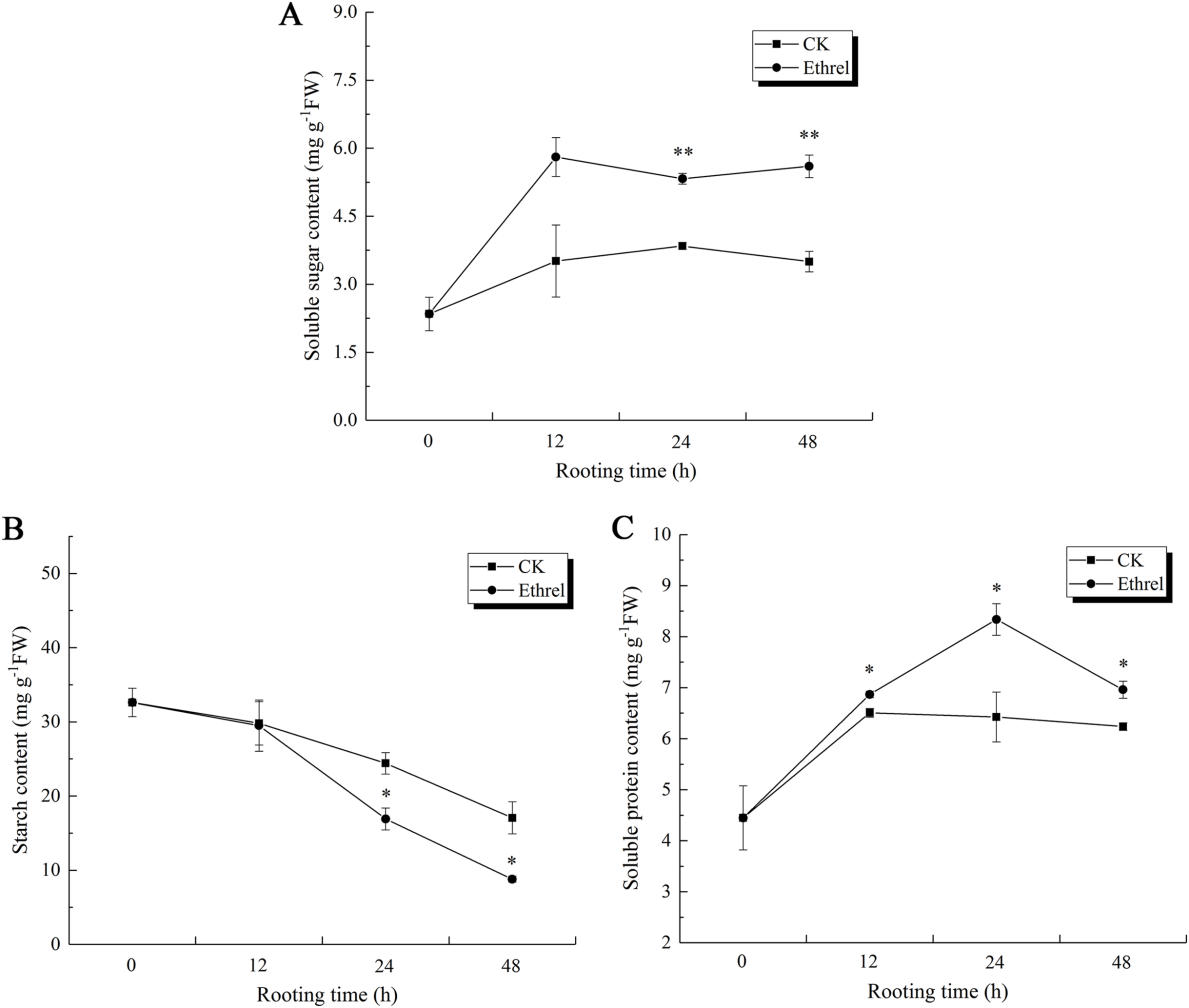
The contents of soluble sugar, starch and soluble protein in cucumber explants. (A) The content of soluble sugar in cucumber explants. (B) The content of starch in cucumber explants. (C) The content of soluble protein in cucumber explants. The value represents the mean ± SE (*n* = 3). Significant differences between treatments are indicated by asterisk (*), “*” represents *P* < 0.05, “**” represents *P* < 0.01.

Taking into consideration that AtpA is also a carbon and energy metabolic protein, the changes in the soluble sugar, starch and total soluble protein content were measured in the hypocotyls during adventitious rooting (Fig. 13). The soluble sugar content in the ethylene-treated plants increased gradually between 0 and 12 h but had declined by 24 h. After that, it showed an increasing trend at 48 h. The highest soluble sugar content was observed in the ethylene treatment, in which it was 66.6% higher than that of the control at 48 h (Fig. 13A).

Starch content showed the same trends in both the control and ethrel treatments (Fig. 13B). After removing the primary root, starch contents decreased over the entire rooting period. At the 24 and 48 h time points, the starch content of explants treated with ethrel were significantly lower (by 30.7% and 48.5%, respectively) than those of the control explants.

In addition, Fig. 13C shows that total soluble proteins in both the control and ethylene treatments reached their highest levels at 24 h and then decreased. Explants treated with ethylene had higher total soluble protein levels than the control. At 24 h, treatment with ethylene had increased total soluble proteins by 12.7% over the levels of the control group.

## Discussion

Ethylene is a plant hormone with numerous physiological functions. The hormone promotes fruit cell expansion and grain maturation, induces flower, leaf and fruit shedding, induces flower bud differentiation, aids dormancy and promotes germination, dwarfing and adventitious root formation (Fischer & Bennett, 1991; Serek, Jones & Reid, 1994; Xu, Li & Qi, 2010; Xu et al., 2017). In the present study, 115 identified proteins were ultimately considered to be differentially expressed adventitious root developmental proteins. Of these, 24 were selected as candidate proteins. The distribution of these 24 DEPs is shown in our association analysis. Recently, ethylene has been shown to have a positive effect on the early induction and expression of adventitious root formation, which may be closely related to auxin activity (Druege, Franken & Hajirezaei, 2016). Another study has shown that ethylene is a stimulant in the early induction and expression stages of adventitious root formation; however, at later periods in the adventitious rooting process, ethylene was revealed to have inhibitory effects, and this is usually strongly associated with endogenous auxin (Da Costa et al., 2013). In this study, a large number of proteins were upregulated during the induction stage following ethylene treatment, and the levels of these newly formed proteins were not as high in the induction phase as in the formation and elongation stages. This indicated that the promotive role of ethylene on adventitious rooting mainly operated at the root induction phase.

To verify the results of iTRAQ analysis, the mRNA expression levels of the 23 DEPs were estimated by qPCR. The results showed that trends in the relative expression levels of the genes encoding 19 of the DEPs were consistent with those of their corresponding DEPs. These proteins are involved in carbohydrate and energy metabolism, amino acid metabolism, stress defense, photosynthesis, transcription and translation, membrane and transporter proteins, ion transport, and protein folding, modification, degradation and location. In addition, we observed that the mRNA levels of four proteins did not correspond to their protein expression patterns, including two amino acid metabolism-related proteins, one transcription- and translation-related protein, and one protein folding, modification, degradation and location-related protein. The difference between the levels of protein and mRNA expression for these proteins may be caused by many factors, such as translational or post-translational regulation (Tian et al., 2004). Therefore, multiple competing factors may influence the regulation of ethylene-induced adventitious root formation.

It is important note that SAM is the precursor of ethylene and polyamine biosynthesis. Among the DEPs in this study, SAMS is a key enzyme in plant metabolism, which catalyzes reactions of polyamines and ethylene biosynthesis. SAMS can also bind with RNA and participate in the regulation of gene expression in vivo (Winkler et al., 2003; Grundy & Henkin, 2004). A study on *Arabidopsis* showed that *SAMS* gene plays an important role in the formation of vascular bundles in the phloem of plants, which directly regulates the formation of root primordia (Pommerrenig et al., 2011; Waduwara-Jayabahu et al., 2012). In addition, *SAMS* was highly expressed in the basal tissue of black locust (*Robinia pseudoacacia* L.) cuttings during adventitious root formation in a study on cuttage, indicating that *SAMS* is closely associated with the rooting process (Quan et al., 2014). In this study, western blot analysis revealed that the abundance of SAMS was markedly elevated by the exogenous application of ethylene in cucumber explants during the phases of root induction and formation. Ethylene synthesis increased at the induction stage and reached its maximum value at the root primordium formation stage, and then decreased at the elongation stage. However, ethylene production at 48 h was higher under the ethrel treatment than under the control conditions, which may be due to the absorption of exogenous ethylene by the plant tissue. This phenomenon indicated that exogenous ethylene induces endogenous ethylene production. The upregulation of endogenous ethylene biosynthesis is the precondition for its regulatory role in plant physiological processes. The high expression of multiple ACO proteins suggests that ethylene biosynthesis is strongly stimulated at the ACC synthesis level (Lei et al., 2018). In higher plants, *ACS* (encoding 1-aminocyclopropane-1-carboxylate synthase, ACS) and *ACO* (encoding 1-aminocyclopropane-1-carboxylate oxidase, ACO) are the key genes in the ethylene biosynthesis pathway. Changes in the expression of these genes could regulate fruit ethylene content and control the ripening stage of fruit (Cara & Giovannoni, 2008). In the present study, the activities of ACO and ACS and the relative expression levels of *ACO1* and *ACS2* showed an increasing trend during the explant rooting process. However, the activities and gene expression levels of ACO and ACS were downregulated when the ethylene inhibitors AVG and AgNO_3_ were applied, indicating that the inhibition of endogenous ethylene synthesis resulted in a significant decrease in ethylene production. In a previous study, transcriptomics of the mango cotyledon revealed that genes associated with adventitious roots included ethylene synthetase-related genes (such as *ACO*), *SAM*, and ethylene receptor gene *ERFs* (Li et al., 2017). The relative expression levels of *ETR1* and *ERS*, both of which were suppressed during the induction and formation stages of adventitious roots, were upregulated by the ethylene inhibitors AVG and AgNO_3_. This may be due to the negative feedback effect of *ETR* on ethylene signal transduction (Hua et al., 1998). Comprehensive analyses of the different rooting stages of cucumber explants showed that ethylene release and the expression of ethylene biosynthesis-related genes varied widely among the different stages of adventitious root development in cucumber explants. Among the three rooting stages, the release of ethylene increased at the induction stage and reached its maximal value at the stage of adventitious root primordium formation. Meanwhile, the enzyme activities of ACO and ACS and the relative expression of their encoding genes were consistent with the trend involved in the release of ethylene.

A previous study found that exogenous ethylene could positively regulate the expression of the ethylene receptor-related gene *ETR1* in *Citrus sinensis*, but that the expression of *ERS1* (ethylene response sensor 1) remained constant (Katz et al., 2004). However, in this study, the mRNA levels of *ETR1* and *ERS* were shown to be downregulated by ethylene treatment, indicating that they were not positively regulated by ethylene. It has been reported that the transcriptional levels of genes in the ethylene receptor gene family can be regulated by ethylene (Hua & Meyerowitz, 1998). Exogenous ethylene significantly enhanced the activity of enzymes and the expression of their encoding genes in the ethylene biosynthesis pathway, and decreased the expression of ethylene receptor genes *ETR1* and *ERS*, indicating that the synthesis of endogenous ethylene as well as the formation of adventitious roots were promoted. Therefore, we speculate that SAMS is directly involved in the regulation of adventitious root development as a precursor to the ethylene synthesis pathway.

Ethylene has gained much attention in recent years in the regulation of photosynthetic capacity (Khan, 2004; Iqbal et al., 2013). Chloroplast ATP synthase (ATP enzyme, CF1-cf 0) catalyzes the main energy-supplying reaction in photosynthesis (Zhang et al., 2018). Although there is no report on the relationship between the ATP synthase subunit a and plant rooting, an increasing number of studies on *atpI*, which encodes ATP synthase subunit a, have been conducted in recent years. ATP synthase, especially its subunit a, is essential for photosynthesis and energy metabolism (Rott et al., 2011). The results of this study have shown that ATP synthase subunit a protein expression in the hypocotyls of cucumber explants was upregulated during the induction and formation stages. The protein expression of ATP synthase subunit a increased at 12 and 24 h, and then decreased at 48 h. When the rooting process began in the cucumber hypocotyl, it probably required more ATP synthase to participate in photosynthesis and energy metabolism after hormone induction. The light capture ability of photosynthesis in plant leaves is reflected by the chlorophyll content, which is key to maintaining normal photosynthesis (Xu, Li & Zhao, 2004). In the present study, the content of chlorophyll in the explants was increased by exogenous ethylene, which could enhance both photosynthesis and chloroplast integrity, thereby providing sufficient energy and carbon sources to support the formation of adventitious roots. On the other hand, during the aging process of plants, ethylene can improve cotyledon senescence and abscission. For example, before etiolation, the ethylene content increased significantly in bean (*Phaseolus vulgaris* L.) cotyledons (Wilhelmová et al., 2004). Ethylene also induced chlorophyll breakdown in *Cucumis sativus* cotyledons during yellowing (Abeles & Dunn, 1989). However, exogenous ethylene improved the chlorophyll content and plant growth of alfalfa (*Medicago sativa* L.) seedlings under NaCl stress (Wang et al., 2020). In the present study, the chlorophyll content was enhanced by treatment with ethylene, indicating that seedling age and growth conditions (stress or mechanical damage) may affect the direction of the effects of ethylene as a regulatory hormone signal.

The chlorophyll fluorescence parameters, including Fv/Fm, ФPS II and qP, were significantly improved by exogenous ethylene, which indicated that the absorption and conversion efficiency of light energy was improved in PS II. These results are similar to earlier findings on cucumber seedlings subjected to drought stress (Chen et al., 2017b). Moreover, exogenous application of ethylene affected not only the light capture capacity of PS II but also the Calvin cycle in the dark reaction stage. The reaction efficiency of the Calvin cycle is the key limiting factor affecting the CO_2_ assimilation efficiency (Dujardyn & Foyer, 1989). In this study, ethylene treatment significantly enhanced the activities of key enzymes involved in the Calvin cycle, including Rubisco, GAPDH, FBA, FBPase and TK. In other words, ethylene promoted the efficiency of the photosynthetic dark reactions by regulating the activities of enzymes related to carbon assimilation and RuBP regeneration in chloroplasts. A study on mini Chinese cabbage showed that the gene expression levels and enzyme activities of rbcL, rbcS, FBA, FBPase and TK were enhanced by the application of an appropriate ammonia-to-nitrate ratio, which led to an increase in the carbohydrate content in the plants and higher tolerance to low light stress (Hu et al., 2017). Studies have shown that the upregulation of genes involved in the Calvin cycle results in an increase in the net photosynthetic rate and enhances plant vegetative growth (Miyagawa, Tamoi & Shigeoka, 2001; Xia et al., 2009). The relative expression levels of the *rbc-L*, *GAPDH*, *FBA* and *TK* genes in ethylene-treated cucumber explants were upregulated at both the induction and formation stages. This indicated that the efficiency of carbon assimilation could be enhanced by ethylene during the early stages of adventitious rooting. Moreover, starch is a common storage compound in plants (Leroy et al., 2019). These results suggest that exogenous ethylene activates the Calvin cycle at both the transcriptional and enzymatic levels, and then enhances the production of assimilates.

Energy metabolism is indispensable for plant growth, development and organogenesis. The formation of adventitious roots requires a large amount of carbon skeletons and energy (Calamar & De Klerk, 2002; Druege, Zerche & Kadner, 2004). Carbohydrates are the principle compounds used for energy storage and transport in plants. In fact, changes in plant carbohydrate content are related to the rooting process (Bakshi & Husen, 2002). Starch hydrolysis is the source of soluble sugars, which can then be exported to heterotrophic tissues, such as the roots, where they are assimilated for growth (MacNeill et al., 2017). Starch hydrolysis in cucumber seedlings may be related to the increase in soluble sugar content. Soluble sugars not only provide the energy for reactions involved in rooting but constitute the source of energy for most metabolic processes. The starch content of ethylene-treated explants was lower than that of control plants during all rooting stages. However, the soluble sugar content was higher than that of the control plants. This might be due to the acceleration of starch hydrolysis in explants under ethylene treatment, which would have provided the necessary energy for cell division and differentiation and increased the rate of energy metabolism in explants during rooting. In teak (*Tectona grandis* L.) cuttings, the soluble protein content was observed to steadily increase but decreased after adventitious roots were formed (Husen, 2008). At the induction stage, soluble protein content increased in explants, which is probably related to the initiation and differentiation of cells. Then, the soluble protein content decreased after 24 h, which indicated that the formation of root primordium might consume a large amount of soluble protein during root growth. In a study involving chrysanthemums (*Dendranthema morifolium*) and marigolds (*Tagetes erecta* L.), exogenous NO and H_2_O_2_ promoted adventitious root formation by increasing soluble sugar and soluble protein content (Liao, Xiao & Zhang, 2010; Liao et al., 2012). Therefore, exogenous ethylene strengthens plant energy metabolism; over the entire rooting process, it enhanced the provision of adequate nutrition and energy for cell division and differentiation.

## Conclusions

The proteome analysis conducted in the current study revealed that among 115 identified proteins, there were 23 proteins associated with the development of adventitious roots in cucumber explants. These were mainly related to intracellular functions such as photosynthesis, transcription and translation, carbon and energy metabolism, and amino acid metabolism. In addition, after treatment with exogenous ethylene, a large amount of endogenous ethylene is released during the root induction and formation phases, and this co-occurs with the enhancement of ACS, ACO and SAMS expression, suggesting that the biosynthesis of endogenous ethylene can be stimulated by exogenous ethylene. The downregulation of *ETR1* and *ERS* expression indicated that *ETR1* and *ERS* play negative regulatory roles in ethylene production. This indicates that ethylene participated in and promoted the formation of adventitious roots in cucumber explants. Moreover, subunit a of ATP synthase was promoted by exogenous ethylene, which led to the enhancement of photosynthesis and the improvement of light energy utilization. Through the application of exogenous ethylene, key enzymes in the Calvin cycle were enhanced to accelerate the CO_2_ assimilation efficiency, which could provide more carbon skeletons and energy to support the development of adventitious roots. In summary, SAMS and AtpA were participants in endogenous ethylene synthesis and photosynthetic energy metabolism during the ethylene-induced adventitious rooting process.

## Supplemental Information

10.7717/peerj.10887/supp-1 Supplemental Information 1 GO classification of the DEPs detected in cucumber explants at 12 h.

10.7717/peerj.10887/supp-2 Supplemental Information 2 GO classification of the DEPs detected in cucumber explants at 24 h.

10.7717/peerj.10887/supp-3 Supplemental Information 3 GO classification of the DEPs detected in cucumber explants at 48 h.

10.7717/peerj.10887/supp-4 Supplemental Information 4 All Differentially expressed proteins during the induction of adventitious roots of cucumber explants by ethylene.

10.7717/peerj.10887/supp-5 Supplemental Information 5 Raw data of qPCR, content of ethylene, sugar, starch, and enzyme activities.

10.7717/peerj.10887/supp-6 Supplemental Information 6 The original pictures of Western blot.

## Additional Information and Declarations

### Data Availability

The following information was supplied regarding data availability: The mass spectrometry proteomics data are available at the ProteomeXchange Consortium via the PRIDE partner repository: PXD016444. The blot pictures are available as a Supplemental File.
