# Supplementary figures and images for: Proteomic analysis reveals key proteins involved in ethylene-induced adventitious root development in cucumber (Cucumis sativus L.)
Source: PeerJ. 2021 Apr 6;9:e10887. doi: 10.7717/peerj.10887 (PMC8034359; doi:10.7717/peerj.10887)

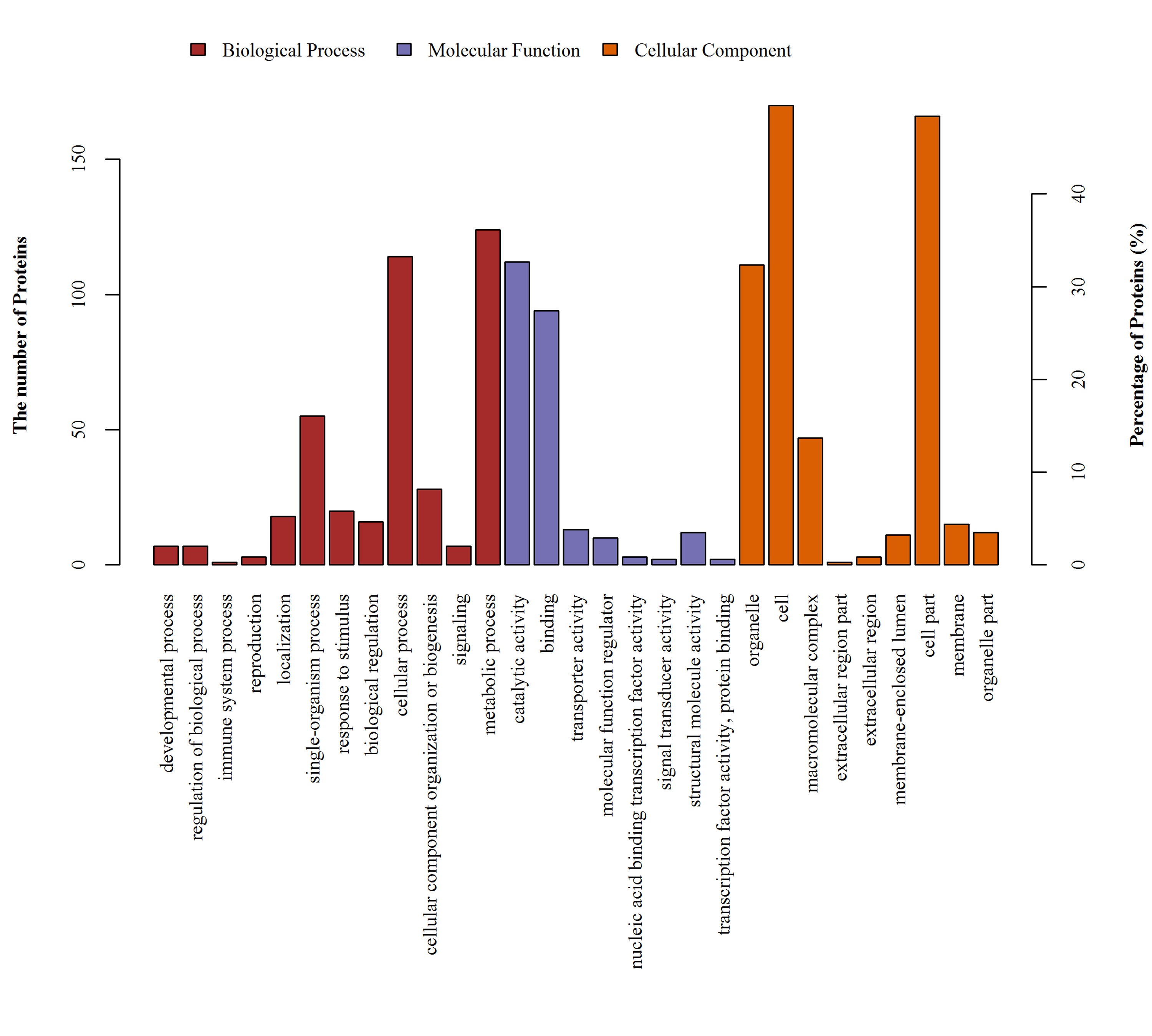

Supplement: Supplemental Information 1 [file peerj-09-10887-s001.png]

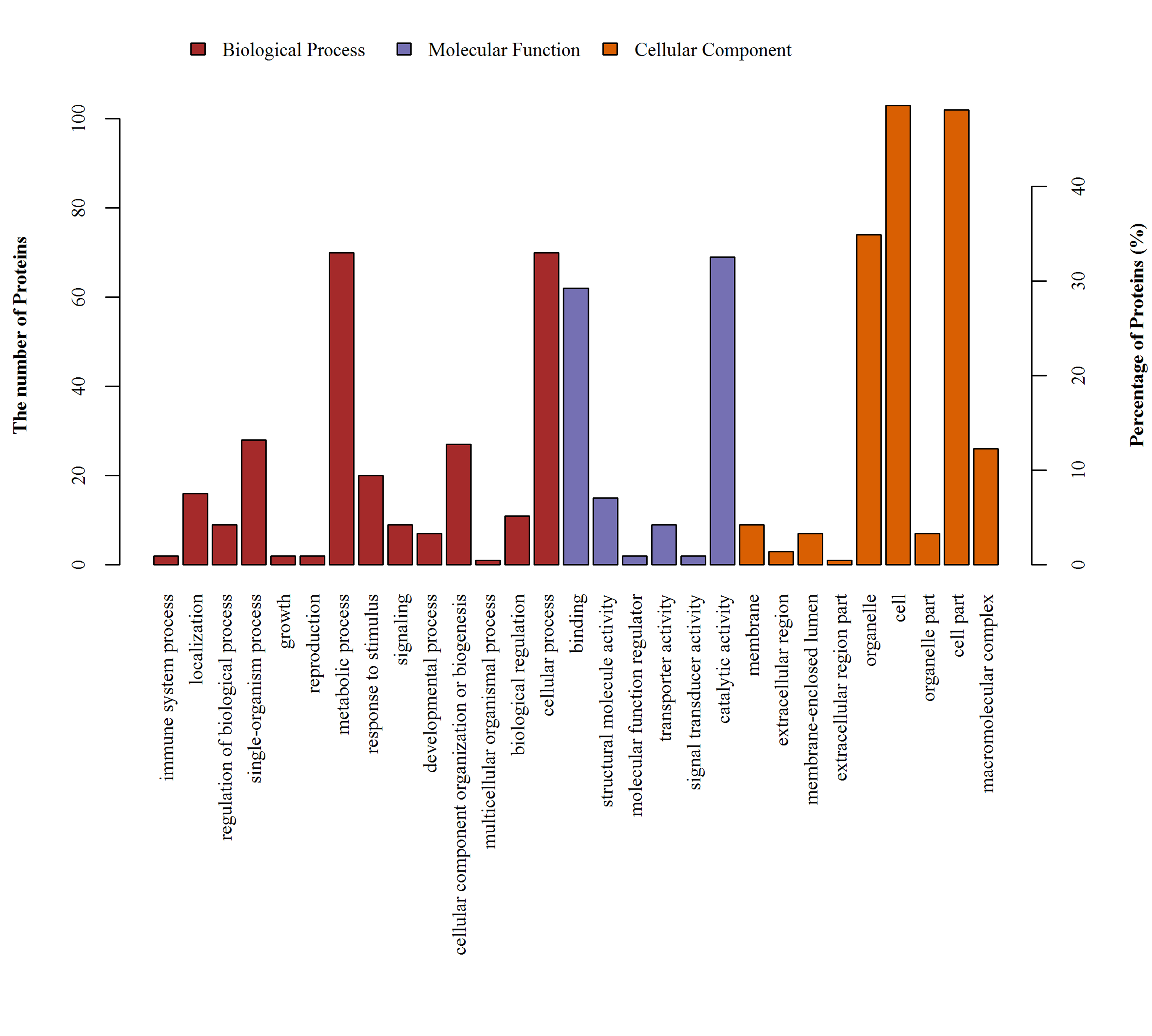

Supplement: Supplemental Information 2 [file peerj-09-10887-s002.png]

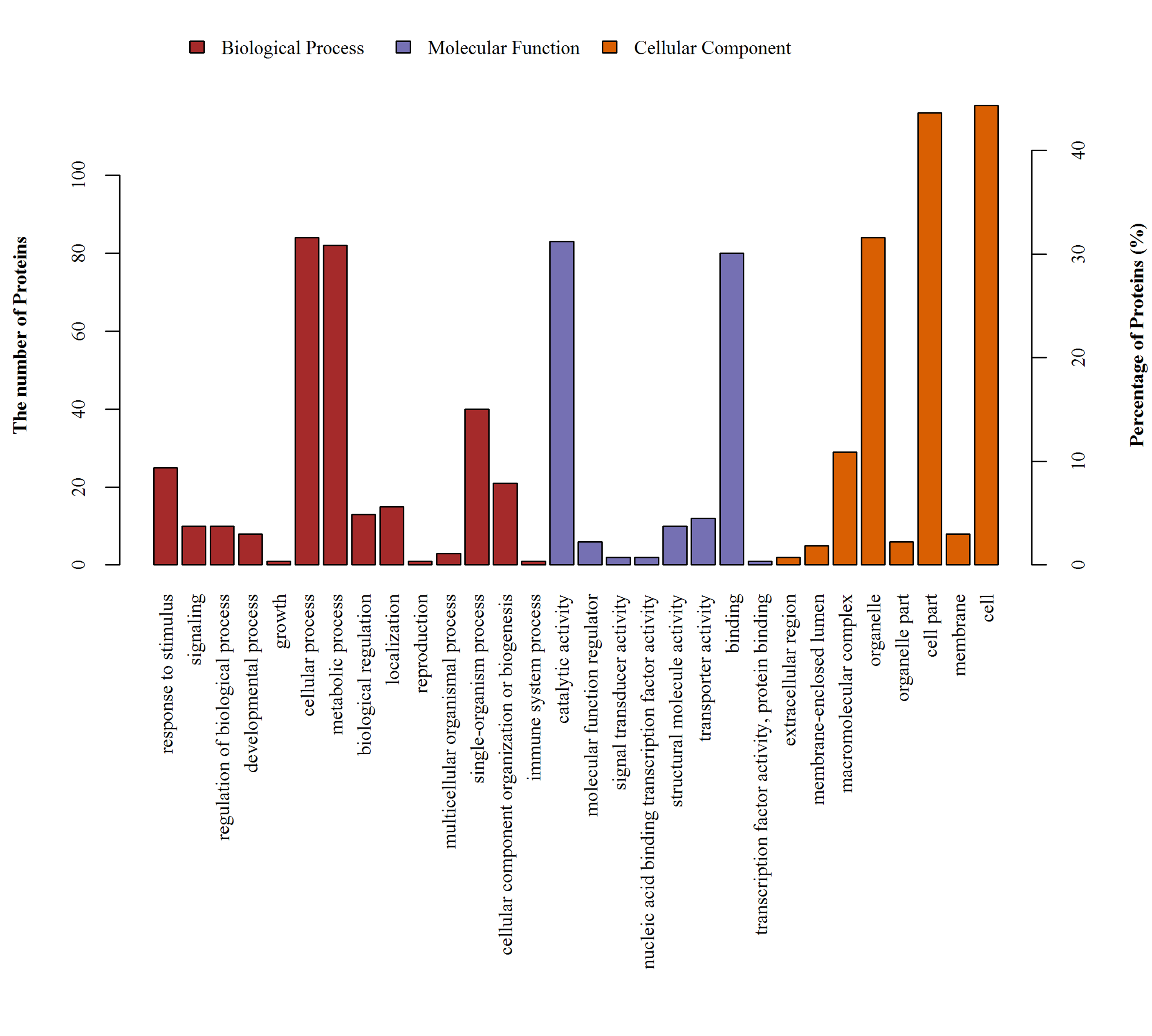

Supplement: Supplemental Information 3 [file peerj-09-10887-s003.png]
