# Supplementary material for: Proteomic analysis reveals key proteins involved in ethylene-induced adventitious root development in cucumber (Cucumis sativus L.)
Source: PeerJ. 2021 Apr 6;9:e10887. doi: 10.7717/peerj.10887 (PMC8034359; doi:10.7717/peerj.10887)
Supplement: Supplemental Information 4 [file peerj-09-10887-s004.docx]

**Table S1 All Differentially expressed proteins during the induction of adventitious roots of cucumber explants by ethylene**

| **Accession** | **Description** | **E12vs C12** | **E24vsC24** | **E48vsC48** |
| --- | --- | --- | --- | --- |
| A0A0A0KIS8 | Mitochondrial dicarboxylate carrier protein | ↑ | ↓ | ↑ |
| A0A0A0K6E1 | Uncharacterized protein | ↑ | ↑ | ↓ |
| G3EIX1 | NADH-ubiquinone oxidoreductase chain 5 | ↑ | ↑ | ↓ |
| A0A0A0L5L0 | Uncharacterized protein | ↑ | ↑ | ↓ |
| A0A0A0K5X0 | S-adenosylmethionine synthase | ↑ | ↑ | ↓ |
| A0A0A0L6I8 | Chlorophyll a-b binding protein, chloroplastic | ↑ | ↑ | ↓ |
| Q4VZP5 | ATP synthase subunit a, chloroplastic | ↑ | ↑ | ↓ |
| A0A0A0KJJ3 | Uncharacterized protein | ↑ | ↑ | ↓ |
| A0A0A0L3T3 | Uncharacterized protein | ↑ | ↓ | ↑ |
| A0A0A0K6I2 | Uncharacterized protein | ↑ | ↓ | ↑ |
| A0A0A0LB48 | Uncharacterized protein | ↑ | ↓ | ↑ |
| A0A0A0LE62 | Uncharacterized protein | ↑ | ↓ | ↑ |
| A0A0A0LM97 | Uncharacterized protein | ↑ | ↓ | ↑ |
| A0A0A0LIP5 | Uncharacterized protein | ↑ | ↓ | ↑ |
| A0A0A0KES3 | Uncharacterized protein | ↑ | ↓ | ↓ |
| A0A0A0K8W2 | Uncharacterized protein | ↓ | ↑ | ↓ |
| A0A0A0LVH4 | Cystatin Hv-CPI6 | ↓ | ↑ | ↓ |
| A0A0A0LMV1 | Uncharacterized protein | ↓ | ↓ | ↑ |
| A0A0A0KR08 | Uncharacterized protein | ↓ | ↓ | ↑ |
| A0A0A0L4I7 | Uncharacterized protein | ↓ | ↓ | ↑ |
| A0A0A0KTT0 | Aspartokinase | ↓ | ↓ | ↑ |
| A0A0A0LGJ0 | Zinc finger protein | ↓ | ↓ | ↑ |
| A0A0A0KSY5 | Uncharacterized protein | ↓ | ↓ | ↑ |
| A0A0A0KD81 | Uncharacterized protein | ↓ | ↓ | ↑ |
| A0A0A0KQF1 | Uncharacterized protein | ↓ | ↓ | ↑ |
| A0A0A0KD16 | Uncharacterized protein | ↓ | ↓ | ↑ |
| A0A0A0KNG7 | Uncharacterized protein | ↓ | ↓ | ↑ |
| A0A0A0KQS8 | Uncharacterized protein | ↓ | ↓ | ↑ |
| A0A0A0KPK3 | Uncharacterized protein | ↓ | ↓ | ↑ |
| A0A0A0LWS6 | Uncharacterized protein | ↓ | ↓ | ↑ |
| A0A0A0K7G4 | Uncharacterized protein | ↓ | ↓ | ↑ |
| A0A0A0KSM7 | Uncharacterized protein | ↓ | ↓ | ↑ |
| A0A0A0LW06 | Uncharacterized protein | ↓ | ↓ | ↑ |
| A0A0A0LU62 | Uncharacterized protein | ↓ | ↓ | ↑ |
| A0A0A0KGQ0 | Uncharacterized protein | ↓ | ↓ | ↑ |
| A0A0A0KTQ0 | Uncharacterized protein | ↓ | ↓ | ↑ |
| A0A0A0KNB9 | DNA-directed RNA polymerase subunit beta | ↓ | ↓ | ↑ |
| A0A0A0KE25 | Uncharacterized protein | ↓ | ↓ | ↑ |
| A0A0A0K7C5 | Uncharacterized protein | ↓ | ↓ | ↑ |
| A0A0A0LGI8 | Uncharacterized protein | ↓ | ↓ | ↑ |
| A0A0A0KDH9 | Uncharacterized protein | ↓ | ↓ | ↓ |
| A0A0A0LJ10 | Glucose-1-phosphate adenylyltransferase | ↑ | ↑ | - |
| A0A0A0LNA5 | Uncharacterized protein | ↑ | ↑ | - |
| A0A0A0KQQ4 | Uncharacterized protein | ↑ | ↑ | - |
| A0A0A0KZY1 | Uncharacterized protein | ↑ | ↑ | - |
| A0A0A0LVU1 | Uncharacterized protein | ↑ | ↓ | - |
| A0A0A0LWD1 | Uncharacterized protein | ↑ | ↓ | - |
| A0A0A0LZK1 | Uncharacterized protein | ↑ | ↓ | - |
| A0A0A0L1Q9 | Uncharacterized protein | ↑ | ↓ | - |
| A0A0A0LPB2 | Uncharacterized protein | ↑ | ↓ | - |
| A0A0A0LM61 | 40S ribosomal protein S12 | ↑ | ↓ | - |
| A0A0A0KAI6 | Uncharacterized protein | ↑ | ↓ | - |
| A0A0A0LJR9 | Uncharacterized protein | ↑ | ↓ | - |
| A0A0A0LMD9 | Uncharacterized protein | ↑ | ↓ | - |
| A0A0A0L5C3 | Uncharacterized protein | ↑ | ↓ | - |
| A0A0A0KLR3 | Uncharacterized protein | ↑ | ↓ | - |
| A0A0A0LDY1 | Uncharacterized protein | ↑ | ↓ | - |
| A0A0A0K6S6 | Pectinesterase | ↑ | ↓ | - |
| A0A0A0K3P3 | Glycosyltransferase | ↓ | ↑ | - |
| A0A0A0LFY5 | Uncharacterized protein | ↓ | ↑ | - |
| A0A0A0LTG7 | Uncharacterized protein | ↓ | ↑ | - |
| A0A0A0KHZ6 | Uncharacterized protein | ↓ | ↑ | - |
| A0A0A0KYE2 | Uncharacterized protein | ↓ | ↑ | - |
| A0A0A0KRH7 | Uncharacterized protein | ↓ | ↑ | - |
| A0A0A0KZP0 | Uncharacterized protein | ↓ | ↓ | - |
| A0A0A0KA76 | Uncharacterized protein | ↓ | ↓ | - |
| A0A0A0L7P7 | Uncharacterized protein | ↓ | ↓ | - |
| A0A0A0KM12 | Uncharacterized protein | ↓ | ↓ | - |
| A0A0A0KDS6 | Uncharacterized protein | ↓ | ↓ | - |
| A0A0A0LJ81 | Uncharacterized protein | ↓ | ↓ | - |
| A0A0A0LPM3 | Uncharacterized protein | ↓ | ↓ | - |
| A0A0A0LNF1 | Uncharacterized protein | ↓ | ↓ | - |
| A0A0A0KTX7 | Folylpolyglutamate synthase | ↓ | ↓ | - |
| A0A0A0KWI0 | Uncharacterized protein | ↓ | ↓ | - |
| A0A0A0LKN6 | Uncharacterized protein | ↓ | ↓ | - |
| A0A0A0LMS7 | Uncharacterized protein | ↑ | - | ↑ |
| A0A0A0KXZ1 | Uncharacterized protein | ↑ | - | ↑ |
| A0A0A0L796 | Uncharacterized protein | ↑ | - | ↑ |
| A0A0A0KQJ3 | Uncharacterized protein | ↑ | - | ↑ |
| A0A0A0KJQ8 | Hexosyltransferase | ↑ | - | ↑ |
| A0A0A0LD99 | Uncharacterized protein | ↑ | - | ↑ |
| A0A0A0KX46 | Uncharacterized protein | ↑ | - | ↑ |
| A0A0A0LGF2 | Uncharacterized protein | ↑ | - | ↑ |
| A0A0A0KX63 | Uncharacterized protein | ↑ | - | ↑ |
| A0A0A0LSV2 | Uncharacterized protein | ↑ | - | ↑ |
| A0A0A0KXU0 | Uncharacterized protein | ↑ | - | ↓ |
| A0A0A0LVN2 | Phloem protein | ↑ | - | ↓ |
| Q8LK68 | 26 kDa phloem lectin (Fragment) 26 kDa | ↑ | - | ↓ |
| A0A0A0LYF4 | Phloem filament protein | ↑ | - | ↓ |
| A0A0A0KAR0 | Uncharacterized protein | ↓ | - | ↑ |
| A0A0A0LFZ4 | Uncharacterized protein | ↓ | - | ↑ |
| A0A0A0LLL7 | Uncharacterized protein | ↓ | - | ↑ |
| A0A0A0KAB9 | Uncharacterized protein | ↓ | - | ↑ |
| A0A0A0KVB1 | Uncharacterized protein | ↓ | - | ↑ |
| A0A0A0KJ36 | Uncharacterized protein | ↓ | - | ↑ |
| A0A0A0L7Q2 | Uncharacterized protein | ↓ | - | ↑ |
| A0A0A0KZV4 | Uncharacterized protein | ↓ | - | ↑ |
| A0A0A0LA49 | Uncharacterized protein | ↓ | - | ↑ |
| A0A0A0K8L0 | Uncharacterized protein | ↓ | - | ↑ |
| A0A0A0KKX3 | Uncharacterized protein | ↓ | - | ↑ |
| A0A0A0LBZ2 | Uncharacterized protein | ↓ | - | ↑ |
| A0A0A0L7K1 | Uncharacterized protein | ↓ | - | ↑ |
| A0A0A0KZU7 | Uncharacterized protein | ↓ | - | ↑ |
| A0A0A0LTV3 | Uncharacterized protein | ↓ | - | ↑ |
| A0A0A0K8X3 | Uncharacterized protein | ↓ | - | ↑ |
| A0A0A0KEL4 | Uncharacterized protein | ↓ | - | ↑ |
| A0A0A0KW06 | Uncharacterized protein | ↓ | - | ↑ |
| A0A0A0LRF9 | Uncharacterized protein | ↓ | - | ↑ |
| A0A0A0LVV1 | Uncharacterized protein | ↓ | - | ↑ |
| A0A0A0LIX9 | Uncharacterized protein | ↓ | - | ↓ |
| A0A0A0LG21 | Uncharacterized protein | ↓ | - | ↓ |
| A0A0A0K342 | Uncharacterized protein | ↓ | - | ↓ |
| A0A0A0LLW1 | Uncharacterized protein | ↓ | - | ↓ |
| A0A0A0LCH8 | Uncharacterized protein | ↓ | - | ↓ |
| A0A0A0LPT2 | Uncharacterized protein | ↓ | - | ↓ |
| A0A0A0LSA1 | Uncharacterized protein | ↓ | - | ↓ |
| A0A0A0LI62 | Methionine aminopeptidase | ↓ | - | ↓ |
| A0A0A0K864 | Uncharacterized protein | ↓ | - | ↓ |
| A0A0A0KRW2 | Uncharacterized protein | ↓ | - | ↓ |
| A0A0A0KS62 | Glycosyltransferase | - | ↑ | ↑ |
| A0A0A0LEJ0 | Uncharacterized protein | - | ↑ | ↑ |
| A0A0A0KWM9 | Uncharacterized protein | - | ↑ | ↓ |
| A0A0A0KQA1 | Uncharacterized protein | - | ↑ | ↓ |
| A0A0A0KL83 | Uncharacterized protein | - | ↑ | ↓ |
| A0A0A0KD45 | Uncharacterized protein | - | ↑ | ↓ |
| A0A0A0KH65 | Uncharacterized protein | - | ↑ | ↓ |
| A0A0A0LGM0 | Uncharacterized protein | - | ↑ | ↓ |
| A0A0A0KZX9 | Uncharacterized protein | - | ↑ | ↓ |
| A0A0A0L7A6 | Uncharacterized protein | - | ↑ | ↓ |
| A0A0A0K6L3 | Uncharacterized protein | - | ↓ | ↑ |
| A0A0A0L4Z2 | Uncharacterized protein | - | ↓ | ↑ |
| A0A0A0KQG4 | Uncharacterized protein | - | ↓ | ↑ |
| A0A0A0LP05 | Uncharacterized protein | - | ↓ | ↑ |
| A0A0A0KSN3 | Uncharacterized protein | - | ↓ | ↑ |
| A0A0A0KEW9 | Serine/threonine-protein phosphatase | - | ↓ | ↑ |
| A0A0A0LXJ0 | Uncharacterized protein | - | ↓ | ↑ |
| A0A0A0L5T4 | Uncharacterized protein | - | ↓ | ↑ |
| A0A0A0KZ10 | Uncharacterized protein | - | ↓ | ↑ |
| A0A0A0M3K7 | Uncharacterized protein | - | ↓ | ↑ |
| A0A0A0K839 | Uncharacterized protein | - | ↓ | ↑ |
| A0A0A0K2X7 | Uncharacterized protein | - | ↓ | ↑ |
| A0A0A0L544 | Uncharacterized protein | - | ↓ | ↑ |
| A0A0A0LTA9 | Uncharacterized protein | - | ↓ | ↑ |
| A0A0A0LYE0 | Uncharacterized protein | - | ↓ | ↑ |
| A0A0A0KZD0 | Uncharacterized protein | - | ↓ | ↑ |
| A0A0A0LG86 | Uncharacterized protein | - | ↓ | ↑ |
| A0A0A0KRQ0 | Uncharacterized protein | - | ↓ | ↑ |
| A0A0A0L6Y8 | Uncharacterized protein | - | ↓ | ↑ |
| A0A0A0KDJ3 | Uncharacterized protein | - | ↓ | ↑ |
| A0A0A0KCB9 | Uncharacterized protein | - | ↓ | ↑ |
| A0A0A0LMY0 | Peroxidase | - | ↓ | ↑ |
| A0A0A0LCY0 | Uncharacterized protein | - | ↓ | ↑ |
| A0A0A0LMJ2 | Uncharacterized protein | - | ↓ | ↑ |
| I1Z8C8 | Chloride channel protein | - | ↓ | ↑ |
| A0A0A0KDY9 | Uncharacterized protein | - | ↓ | ↑ |
| A0A0A0L1C7 | Uncharacterized protein | - | ↓ | ↑ |
| A0A0A0KAK4 | Uncharacterized protein | - | ↓ | ↑ |
| A0A0A0L816 | DNA-directed RNA polymerase subunit | - | ↓ | ↑ |
| A0A0A0LUX3 | Uncharacterized protein | - | ↓ | ↑ |
| A0A0A0LK47 | Uncharacterized protein | - | ↓ | ↑ |
| A0A0A0LUK0 | Uncharacterized protein | - | ↓ | ↑ |
| A0A0A0LHY9 | Uncharacterized protein | - | ↓ | ↑ |
| A0A0A0LZP1 | Uncharacterized protein | - | ↓ | ↑ |
| A0A0A0KEV3 | Uncharacterized protein | - | ↓ | ↑ |
| A0A0A0K378 | Uncharacterized protein | - | ↓ | ↓ |
| A0A0A0KS45 | Uncharacterized protein | - | ↓ | ↓ |
| A0A0A0LFW9 | Uncharacterized protein | ↑ | - | - |
| A0A0A0L3Z5 | Uncharacterized protein | ↑ | - | - |
| A0A0A0LX15 | Uncharacterized protein | ↑ |  |  |
| A0A0A0LC13 | Uncharacterized protein | ↑ | - | - |
| A0A0A0KZC6 | Uncharacterized protein | ↑ | - | - |
| A0A0A0LSA7 | Uncharacterized protein | ↑ | - | - |
| Q8S3W3 | Phenylalanine ammonia lyase 1 (Fragment) | ↑ | - | - |
| A0A0A0LTX0 | Uncharacterized protein | ↑ | - | - |
| A0A0A0KU77 | Uncharacterized protein | ↑ | - | - |
| A0A0A0LDQ7 | Uncharacterized protein | ↑ | - | - |
| A0A0A0LRJ4 | Uncharacterized protein | ↑ | - | - |
| A0A0A0KES9 | Uncharacterized protein | ↑ | - | - |
| A0A0A0LNF5 | Uncharacterized protein | ↑ | - | - |
| A0A0A0M0W2 | Uncharacterized protein | ↑ | - | - |
| A0A0A0KE11 | Uncharacterized protein | ↑ | - | - |
| A0A0A0KHG3 | Uncharacterized protein | ↑ | - | - |
| A0A0A0K5R9 | Uncharacterized protein | ↑ | - | - |
| A0A0A0LCJ1 | Uncharacterized protein | ↑ | - | - |
| A0A0A0KHV5 | Pectinesterase | ↑ | - | - |
| A0A0A0KA58 | Uncharacterized protein | ↑ | - | - |
| A0A0A0K7M8 | Uncharacterized protein | ↑ | - | - |
| A0A0A0LXG6 | Uncharacterized protein | ↑ | - | - |
| A0A0A0L8H7 | Uncharacterized protein | ↑ | - | - |
| Q4VZJ2 | 30S ribosomal protein S12, chloroplastic | ↑ | - | - |
| A0A0A0L4N3 | Uncharacterized protein | ↑ | - | - |
| A0A0A0L5H3 | Uncharacterized protein | ↑ | - | - |
| A0A0A0LPD9 | Cysteine protease | ↑ | - | - |
| A0A0A0LNS6 | Uncharacterized protein | ↑ | - | - |
| A0A0A0LWL2 | Uncharacterized protein | ↑ | - | - |
| A0A0A0K9I4 | Uncharacterized protein | ↑ | - | - |
| A0A0A0LUI0 | Uncharacterized protein | ↑ | - | - |
| A0A0A0K6J5 | Uncharacterized protein | ↑ | - | - |
| A0A0A0LUH4 | Uncharacterized protein | ↑ | - | - |
| A0A0A0KA08 | 40S ribosomal protein S12 | ↑ | - | - |
| A0A0A0KIT4 | Uncharacterized protein | ↑ | - | - |
| A0A0A0KYQ6 | Carbon catabolite repressor protein | ↑ | - | - |
| A0A0A0KP40 | Peptidyl-prolyl cis-trans isomerase | ↑ | - | - |
| A0A0A0LT87 | Uncharacterized protein | ↑ | - | - |
| A0A0A0KTT9 | Uncharacterized protein | ↑ | - | - |
| A0A0A0M324 | DnaJ | ↑ | - | - |
| A0A0A0LD22 | Histone H2A | ↑ | - | - |
| A0A0A0K7J0 | Uncharacterized protein | ↑ | - | - |
| A0A0A0KNA3 | Uncharacterized protein | ↑ | - | - |
| A0A0A0LEU5 | Uncharacterized protein | ↑ | - | - |
| A0A0A0KLC6 | Uncharacterized protein | ↑ | - | - |
| A0A0A0K5X6 | Uncharacterized protein | ↑ | - | - |
| A0A0A0KMA7 | Uncharacterized protein | ↑ | - | - |
| A0A0A0L4B4 | Uncharacterized protein | ↑ | - | - |
| A0A0A0KBU9 | Uncharacterized protein | ↑ | - | - |
| A0A0A0LW64 | 40S ribosomal protein S25 | ↑ | - | - |
| A0A0A0LNG6 | Uncharacterized protein | ↑ | - | - |
| A0A0A0LPC9 | Uncharacterized protein | ↑ | - | - |
| A0A0A0K6B8 | Uncharacterized protein | ↑ | - | - |
| A0A0A0K7B3 | Phloem filament protein | ↑ | - | - |
| A0A0A0KWH2 | Uncharacterized protein | ↑ | - | - |
| A0A0A0LK55 | Uncharacterized protein | ↑ | - | - |
| A0A0A0KHF4 | Uncharacterized protein | ↑ | - | - |
| A0A0A0KF65 | Uncharacterized protein | ↑ | - | - |
| A0A0A0LQJ8 | Uncharacterized protein | ↑ | - | - |
| A0A0A0LG61 | Uncharacterized protein | ↑ | - | - |
| A0A0A0LKC1 | Uncharacterized protein | ↑ | - | - |
| A0A0A0KPV4 | Uncharacterized protein | ↑ | - | - |
| A0A0A0LPQ6 | Uncharacterized protein | ↑ | - | - |
| A0A0A0KDS5 | Uncharacterized protein | ↑ | - | - |
| A0A0A0KQB1 | Uncharacterized protein | ↑ | - | - |
| A0A0A0LBL1 | Uncharacterized protein | ↑ | - | - |
| A0A0A0KHE9 | Uncharacterized protein | ↑ | - | - |
| Q6UNT3 | Hypersensitive-induced response protein | ↑ | - | - |
| A0A0A0LUM1 | Uncharacterized protein | ↑ | - | - |
| A0A0A0L0V6 | Uncharacterized protein | ↑ | - | - |
| A0A0A0KBP8 | Poly(A)-binding protein C-terminal interacting protein 6 | ↑ | - | - |
| A0A0A0KXV7 | Uncharacterized protein | ↑ | - | - |
| A0A0A0M1Q8 | Uncharacterized protein | ↑ | - | - |
| A0A0A0LE91 | Uncharacterized protein | ↑ | - | - |
| A0A0A0L2K6 | Uncharacterized protein | ↑ | - | - |
| A0A0A0KZ48 | Uncharacterized protein | ↑ | - | - |
| A0A0A0LC11 | Uncharacterized protein | ↑ | - | - |
| A0A0A0KHL0 | Uncharacterized protein | ↑ | - | - |
| A0A0A0L2W5 | Uncharacterized protein | ↑ | - | - |
| A0A0A0L9J7 | 60S ribosomal protein L6 | ↑ | - | - |
| P00293 | Plastocyanin | ↑ | - | - |
| A0A0A0LBV8 | Uncharacterized protein | ↑ | - | - |
| A0A0A0KU96 | Uncharacterized protein | ↑ | - | - |
| V5RFY5 | Plasma intrinsic protein 1-2 | ↑ | - | - |
| A0A0A0L511 | Uncharacterized protein | ↑ | - | - |
| A0A0A0KP38 | Global transcription factor group | ↑ | - | - |
| A0A0A0LNR7 | Uncharacterized protein | ↑ | - | - |
| A0A0A0KXH3 | Uncharacterized protein | ↑ | - | - |
| A0A0A0KMZ9 | Uncharacterized protein | ↑ | - | - |
| A0A0A0LAX1 | Major latex protein | ↑ | - | - |
| A0A0A0KZA2 | Potassium transporter | ↑ | - | - |
| A0A0A0KQG9 | Uncharacterized protein | ↑ | - | - |
| A0A0A0KIJ8 | Uncharacterized protein | ↑ | - | - |
| A0A0A0L237 | Uncharacterized protein | ↑ | - | - |
| A0A0A0L4W3 | Uncharacterized protein | ↑ | - | - |
| Q9SLQ8 | Oxygen-evolving enhancer protein 2, chloroplastic | ↑ | - | - |
| A0A0A0LQW2 | Uncharacterized protein | ↑ | - | - |
| A0A0A0L6P8 | Uncharacterized protein | ↑ | - | - |
| A0A0A0KI44 | Mitochondrial pyruvate carrier | ↑ | - | - |
| A0A0A0KTT1 | Uncharacterized protein | ↑ | - | - |
| A0A0A0LWS0 | GTP-binding nuclear protein | ↑ | - | - |
| A0A0A0KQS9 | Uncharacterized protein | ↑ | - | - |
| A0A0A0K1C2 | Uncharacterized protein | ↑ | - | - |
| A0A0A0L3W3 | Uncharacterized protein | ↑ | - | - |
| A0A0A0K7X7 | Uncharacterized protein | ↑ | - | - |
| A0A0A0KWR6 | Uncharacterized protein | ↑ | - | - |
| A0A0A0K9A2 | Uncharacterized protein | ↑ | - | - |
| A0A0A0KVS5 | Uncharacterized protein | ↑ | - | - |
| A0A0A0LFP5 | Uncharacterized protein | ↑ | - | - |
| A0A0A0LSX7 | Uncharacterized protein | ↑ | - | - |
| A0A0A0KMB5 | Uncharacterized protein | ↑ | - | - |
| A0A0A0LAI0 | Uncharacterized protein | ↑ | - | - |
| A0A0A0LER0 | Uncharacterized protein | ↑ | - | - |
| A0A0A0KDH7 | Uncharacterized protein | ↑ | - | - |
| A0A0A0K0W6 | Uncharacterized protein | ↑ | - | - |
| A0A0A0LTZ1 | Uncharacterized protein | ↑ | - | - |
| A0A0A0K565 | Chlorophyll a-b binding protein, chloroplastic | ↑ | - | - |
| A0A0A0LAY6 | Uncharacterized protein | ↑ | - | - |
| A0A0A0KXK5 | Uncharacterized protein | ↑ | - | - |
| A0A0A0L8F6 | Uncharacterized protein | ↑ | - | - |
| A0A0A0LI72 | Uncharacterized protein | ↑ | - | - |
| A0A0A0LYU4 | GRIP and coiled-coil domain-containing protein | ↑ | - | - |
| A0A0A0LVP5 | Uncharacterized protein | ↑ | - | - |
| A0A0A0KKA0 | AMP dependent CoA ligase | ↑ | - | - |
| A0A0A0LAW4 | Uncharacterized protein | ↑ | - | - |
| A0A0A0KPK8 | Uncharacterized protein | ↑ | - | - |
| Q4VZH7 | Photosystem II reaction center protein L | ↓ | - | - |
| A0A0A0LN39 | Uncharacterized protein | ↓ | - | - |
| A0A0A0KBA9 | Uncharacterized protein | ↓ | - | - |
| A0A0A0LP91 | Uncharacterized protein | ↓ | - | - |
| A0A0A0KHD8 | Uncharacterized protein | ↓ | - | - |
| A0A0A0KNR6 | Uncharacterized protein | ↓ | - | - |
| A0A0A0KLI1 | Uncharacterized protein | ↓ | - | - |
| A0A0A0KU52 | Protein CLP1 homolog | ↓ | - | - |
| A0A0A0KWC7 | Glutamate dehydrogenase | ↓ | - | - |
| Q4VZJ0 | Protein PsbN | ↓ | - | - |
| A0A0A0L702 | Uncharacterized protein | ↓ | - | - |
| A0A0A0LAB5 | Uncharacterized protein | ↓ | - | - |
| A0A0A0L1W1 | Uncharacterized protein | ↓ | - | - |
| A0A0A0KKL1 | Uncharacterized protein | ↓ | - | - |
| A8JP99 | Plasma membrane ATPase | ↓ | - | - |
| A0A0A0KZ33 | Uncharacterized protein | ↓ | - | - |
| A0A0A0LGA1 | Uncharacterized protein | ↓ | - | - |
| A0A0A0L7Q1 | ATP-dependent (S)-NAD(P)H-hydrate dehydratase | ↓ | - | - |
| A0A0A0L8T5 | Uncharacterized protein | ↓ | - | - |
| A0A0A0LXS7 | Uncharacterized protein | ↓ | - | - |
| A0A0A0LN01 | Uncharacterized protein | ↓ | - | - |
| A0A0A0K3B5 | Uncharacterized protein | ↓ | - | - |
| A0A0A0L845 | Uncharacterized protein | ↓ | - | - |
| A0A0A0KGH2 | Uncharacterized protein | ↓ | - | - |
| A0A0A0KU93 | Uncharacterized protein | ↓ | - | - |
| A0A0A0LCC2 | Uncharacterized protein | ↓ | - | - |
| A0A0A0LGF0 | Uncharacterized protein | ↓ | - | - |
| A0A0A0LF70 | Uncharacterized protein | ↓ | - | - |
| A0A0A0LTA7 | Uncharacterized protein | ↓ | - | - |
| A0A0A0L649 | Uncharacterized protein | ↓ | - | - |
| A0A0A0LT14 | Uncharacterized protein | ↓ | - | - |
| A0A0A0KNR5 | Uncharacterized protein | ↓ | - | - |
| A0A0A0K4W1 | Uncharacterized protein | ↓ | - | - |
| A0A0A0KH96 | Uncharacterized protein | ↓ | - | - |
| A0A0A0LC56 | Uncharacterized protein | ↓ | - | - |
| A0A0A0LUF4 | Uncharacterized protein | ↓ | - | - |
| A0A0A0L8A1 | Uncharacterized protein | ↓ | - | - |
| A0A0A0K9Z2 | Uncharacterized protein | ↓ | - | - |
| A0A0A0KR09 | Uncharacterized protein | ↓ | - | - |
| A0A0A0KP21 | Oleosin | ↓ | - | - |
| A0A0A0K8L9 | Uncharacterized protein | ↓ | - | - |
| A0A0A0L3N6 | Uncharacterized protein | ↓ | - | - |
| A0A0A0M2E7 | Uncharacterized protein | ↓ | - | - |
| A0A0A0LRH5 | Uncharacterized protein | ↓ | - | - |
| A0A0A0KNA1 | Uncharacterized protein | ↓ | - | - |
| A0A0A0LEP9 | Uncharacterized protein | ↓ | - | - |
| A0A0A0L5X9 | 4-hydroxy-4-methyl-2-oxoglutarate aldolase | ↓ | - | - |
| A0A0A0LBD8 | Uncharacterized protein | ↓ | - | - |
| A0A0A0LZT1 | Uncharacterized protein | ↓ | - | - |
| A0A0A0KK55 | Uncharacterized protein | ↓ | - | - |
| A0A0A0L5I1 | Uncharacterized protein | ↓ | - | - |
| A0A0A0KR19 | Uncharacterized protein | ↓ | - | - |
| A0A0A0KL78 | ATP synthase gamma chain | ↓ | - | - |
| A0A0A0KCX2 | Uncharacterized protein | ↓ | - | - |
| A0A0A0LEP8 | Uncharacterized protein | ↓ | - | - |
| A0A0A0LMF7 | Uncharacterized protein | ↓ | - | - |
| A0A0A0LC78 | ATP-dependent Clp protease proteolytic subunit | ↓ | - | - |
| A0A0A0LI51 | Uncharacterized protein | ↓ | - | - |
| A0A0A0K3W8 | Uncharacterized protein | ↓ | - | - |
| A0A0A0L703 | Uncharacterized protein | ↓ | - | - |
| A0A0A0LZ49 | Uncharacterized protein | ↓ | - | - |
| A0A0A0LX29 | Uncharacterized protein | ↓ | - | - |
| A0A0A0LCU0 | Uncharacterized protein | ↓ | - | - |
| A0A0A0L5C5 | Uncharacterized protein | ↓ | - | - |
| A0A0A0KDI2 | Uncharacterized protein | ↓ | - | - |
| A0A0A0L249 | Uncharacterized protein | ↓ | - | - |
| A0A0A0L619 | Uncharacterized protein | ↓ | - | - |
| A0A0A0KSE6 | Uncharacterized protein | ↓ | - | - |
| A0A0A0KKB4 | Uncharacterized protein | ↓ | - | - |
| A0A0A0KC20 | Cysteine proteinase inhibitor | ↓ | - | - |
| A0A0A0LS48 | Uncharacterized protein | ↓ | - | - |
| A0A0A0LD28 | Uncharacterized protein | ↓ | - | - |
| A0A0A0K6P5 | Uncharacterized protein | ↓ | - | - |
| A0A0A0KSH0 | Uncharacterized protein | ↓ | - | - |
| A0A0A0K5Z9 | Uncharacterized protein | ↓ | - | - |
| A0A0A0K4K4 | Acyl-[acyl-carrier-protein] hydrolase | ↓ | - | - |
| A0A0A0KBF0 | Uncharacterized protein | ↓ | - | - |
| A0A0A0KUF5 | Uncharacterized protein | ↓ | - | - |
| A0A0A0M0N2 | Uncharacterized protein | ↓ | - | - |
| A0A0A0K634 | Uncharacterized protein | ↓ | - | - |
| A0A0A0LPS9 | Uncharacterized protein | ↓ | - | - |
| A0A0A0KQF0 | Chloroplast small heat shock protein class I | ↓ | - | - |
| A0A0A0KHN9 | Uncharacterized protein | ↓ | - | - |
| A0A0A0L5F1 | Uncharacterized protein | ↓ | - | - |
| A0A0A0LPY6 | Uncharacterized protein | ↓ | - | - |
| A0A0A0LD74 | Uncharacterized protein | ↓ | - | - |
| A0A0A0L913 | Uncharacterized protein | ↓ | - | - |
| B0F832 | Eukaryotic initiation factor iso4E | ↓ | - | - |
| A0A0A0LNN6 | Uncharacterized protein | ↓ | - | - |
| A0A0A0LRX4 | Uncharacterized protein | ↓ | - | - |
| A0A0A0LRM4 | Uncharacterized protein | ↓ | - | - |
| Q4VZH4 | Photosystem I assembly protein Ycf3 | ↓ | - | - |
| A0A0A0L709 | Uncharacterized protein | ↓ | - | - |
| A0A0A0L928 | Uncharacterized protein | ↓ | - | - |
| A0A0A0K9P5 | Uncharacterized protein | ↓ | - | - |
| A0A0A0KC19 | Cytochrome P450 | ↓ | - | - |
| A0A0A0KZ67 | Uncharacterized protein | ↓ | - | - |
| A0A0A0LJY3 | Eukaryotic translation initiation factor 6 | ↓ | - | - |
| A0A0A0L1T6 | Uncharacterized protein | - | ↑ | - |
| A0A0A0L362 | Uncharacterized protein | - | ↑ | - |
| A0A0A0KV61 | Uncharacterized protein | - | ↑ | - |
| A0A0A0LP55 | Uncharacterized protein | - | ↑ | - |
| A0A0A0KC46 | Uncharacterized protein | - | ↑ | - |
| A0A0A0K6E3 | Uncharacterized protein | - | ↑ | - |
| A0A0A0KBT8 | Uncharacterized protein | - | ↑ | - |
| A0A0A0L473 | Uncharacterized protein | - | ↑ | - |
| Q4VZH6 | Cytochrome b559 subunit beta | - | ↑ | - |
| A0A0A0KYQ9 | Uncharacterized protein | - | ↑ | - |
| Q96398 | Chromoplast-specific carotenoid-associated protein, chromoplastic | - | ↑ | - |
| A0A0A0L080 | Uncharacterized protein | - | ↑ | - |
| A0A0A0K5Z0 | Carboxypeptidase | - | ↑ | - |
| A0A0A0KU38 | Ribosomal protein L15 | - | ↑ | - |
| A0A0A0M0C4 | Uncharacterized protein | - | ↑ | - |
| A0A0A0LQP6 | Cytochrome P450 | - | ↑ | - |
| A0A0A0LEI9 | Uncharacterized protein | - | ↑ | - |
| A0A0A0L385 | Uncharacterized protein | - | ↑ | - |
| A0A0A0KW41 | Uncharacterized protein | - | ↑ | - |
| A0A0A0KCX6 | Uncharacterized protein | - | ↑ | - |
| A0A0A0KWK4 | Uncharacterized protein | - | ↑ | - |
| A0A0A0KRR5 | Uncharacterized protein | - | ↑ | - |
| A0A0A0KHQ6 | Uncharacterized protein | - | ↑ | - |
| P42051 | Photosystem I reaction center subunit psaK, chloroplastic (Fragment) | - | ↑ | - |
| A0A0A0KCU9 | Basic blue protein | - | ↑ | - |
| A0A0A0K9T8 | Uncharacterized protein | - | ↑ | - |
| A0A0A0LDK7 | Uncharacterized protein | - | ↑ | - |
| A0A0A0KIU7 | Uncharacterized protein | - | ↑ | - |
| A0A0A0LX33 | Uncharacterized protein | - | ↑ | - |
| A0A0A0KDE7 | Uncharacterized protein | - | ↑ | - |
| A0A0A0KUY0 | Protein translocase subunit SecA | - | ↑ | - |
| A0A0A0KQ62 | Uncharacterized protein | - | ↑ | - |
| A0A0A0LI88 | Uncharacterized protein | - | ↑ | - |
| A0A0A0KNC8 | Uncharacterized protein | - | ↑ | - |
| A0A0A0LQ87 | Uncharacterized protein | - | ↑ | - |
| A0A0A0L6Z3 | Flavin-containing monooxygenase | - | ↓ |  |
| A0A0A0KUX2 | Uncharacterized protein | - | ↓ | - |
| A0A0A0KCH3 | Uncharacterized protein | - | ↓ | - |
| A0A0A0LKY5 | Uncharacterized protein | - | ↓ | - |
| A0A0A0KA69 | Uncharacterized protein | - | ↓ | - |
| A0A0A0LWN4 | Uncharacterized protein | - | ↓ | - |
| A0A0A0LRI5 | Inositol-tetrakisphosphate 1-kinase | - | ↓ | - |
| A0A0A0KRT9 | Uncharacterized protein | - | ↓ | - |
| A0A0A0L368 | Chalcone-flavonone isomerase family protein | - | ↓ | - |
| A0A0A0KJR1 | Uncharacterized protein | - | ↓ | - |
| A0A0A0L048 | Cytokinin riboside 5'-monophosphate phosphoribohydrolase | - | ↓ | - |
| A0A0A0K5B9 | Uncharacterized protein | - | ↓ | - |
| A0A0A0LGP2 | Uncharacterized protein | - | ↓ | - |
| A0A0A0LIE8 | Uncharacterized protein | - | ↓ | - |
| A0A0A0LHZ5 | Protein kinase | - | ↓ | - |
| A0A0A0L7R4 | Uncharacterized protein | - | ↓ | - |
| A0A0A0L688 | Uncharacterized protein | - | ↓ | - |
| A0A0A0LGN2 | Uncharacterized protein | - | ↓ | - |
| A0A0A0LH02 | Uncharacterized protein | - | ↓ | - |
| Q4VZN5 | 30S ribosomal protein S14, chloroplastic | - | ↓ | - |
| A0A0A0KIR1 | Uncharacterized protein | - | ↓ | - |
| A0A0A0LML1 | Uncharacterized protein | - | ↓ | - |
| A0A0A0KYP0 | Phloem lectin | - | ↓ | - |
| A0A0A0K468 | Uncharacterized protein | - | ↓ | - |
| A0A0A0L5X7 | Uncharacterized protein | - | ↓ | - |
| A0A0A0L2M7 | Uncharacterized protein | - | ↓ | - |
| A0A0A0LGI2 | Glycosyltransferase | - | ↓ | - |
| A0A0A0LDA2 | Uncharacterized protein | - | ↓ | - |
| A0A0A0LJ55 | Uncharacterized protein | - | ↓ | - |
| A0A0A0K3Z5 | Peroxidase | - | ↓ | - |
| A0A0A0LN89 | Uncharacterized protein | - | ↓ | - |
| A0A0A0K8S0 | Uncharacterized protein | - | ↓ | - |
| A0A0A0KAM9 | Uncharacterized protein | - | ↓ | - |
| A0A0A0KGA7 | Uncharacterized protein | - | ↓ | - |
| A0A0A0L475 | Uncharacterized protein | - | ↓ | - |
| A0A0A0K651 | Uncharacterized protein | - | ↓ | - |
| A0A0A0KIM3 | Uncharacterized protein | - | ↓ | - |
| A0A0A0L268 | Uncharacterized protein | - | ↓ | - |
| A0A0A0KE63 | Uncharacterized protein | - | ↓ | - |
| A0A0A0KXH5 | Uncharacterized protein | - | ↓ | - |
| A0A0A0LJ58 | Uncharacterized protein | - | ↓ | - |
| A0A0A0LK87 | Diacylglycerol kinase | - | ↓ | - |
| A0A0A0K7R9 | Uncharacterized protein | - | ↓ | - |
| A0A0A0KI82 | Uncharacterized protein | - | ↓ | - |
| A0A0A0KKK2 | Uncharacterized protein | - | ↓ | - |
| A0A0A0L546 | Thiamine thiazole synthase, chloroplastic | - | ↓ | - |
| A0A0A0LMU6 | Uncharacterized protein | - | ↓ | - |
| A0A0A0KDW1 | Uncharacterized protein | - | ↓ | - |
| A0A0A0L3P2 | Uncharacterized protein | - | ↓ | - |
| A0A0A0M0L4 | Uncharacterized protein | - | ↓ | - |
| A0A0A0K905 | Uncharacterized protein | - | ↓ | - |
| A0A0A0L2I9 | Tubulin beta chain | - | ↓ | - |
| A0A0A0LSU4 | Uncharacterized protein | - | ↓ | - |
| A0A0A0LZK4 | Uncharacterized protein | - | ↓ | - |
| A0A0A0LAW7 | Uncharacterized protein | - | ↓ | - |
| A0A0A0L2Y0 | Uncharacterized protein | - | ↓ | - |
| A0A0A0LQX2 | Uncharacterized protein | - | ↓ | - |
| A0A0A0LKQ2 | Nucleolar GTP-binding protein 1 | - | ↓ | - |
| A0A0A0KJB6 | Histone H1 | - | ↓ | - |
| A0A0A0LHP0 | RuvB-like helicase | - | ↓ | - |
| A0A0A0KGX7 | Purple acid phosphatase | - | ↓ | - |
| A0A0A0LLV0 | Uncharacterized protein | - | - | ↑ |
| A0A0A0LEN6 | Uncharacterized protein | - | - | ↑ |
| A0A0A0K9D7 | Uncharacterized protein | - | - | ↑ |
| A0A0A0LUZ0 | Uncharacterized protein | - | - | ↑ |
| A0A0A0KWB2 | Oleosin | - | - | ↑ |
| A0A0A0KJ06 | Uncharacterized protein | - | - | ↑ |
| A0A0A0K515 | U1 small nuclear ribonucleoprotein C | - | - | ↑ |
| A0A0A0KLN7 | Uncharacterized protein | - | - | ↑ |
| A0A0A0M268 | Uncharacterized protein | - | - | ↑ |
| A0A0A0K7B5 | Uncharacterized protein | - | - | ↑ |
| A0A0A0LGB3 | Uncharacterized protein | - | - | ↑ |
| A0A0A0KUC3 | Uncharacterized protein |  |  | ↑ |
| A0A0A0LFC7 | Uncharacterized protein |  |  | ↑ |
| A0A0A0LWH4 | Uncharacterized protein |  |  | ↑ |
| A0A0A0L4U6 | Ferredoxin-1 | - | - | ↑ |
| A0A0A0KRU8 | Uncharacterized protein | - | - | ↑ |
| A0MCW3 | Pathogen induced 4 protein | - | - | ↑ |
| A0A0A0L4Y7 | Uncharacterized protein | - | - | ↑ |
| A0A0A0KSP5 | Uncharacterized protein | - | - | ↑ |
| A0A0A0KZF1 | Uncharacterized protein | - | - | ↑ |
| A0A0A0KJX7 | Uncharacterized protein | - | - | ↑ |
| A0A0A0L1B7 | Uncharacterized protein |  |  | ↑ |
| A0A0A0KUE5 | Uncharacterized protein |  |  | ↑ |
| A0A0A0L9A7 | Uncharacterized protein |  |  | ↑ |
| A0A0A0K632 | Uncharacterized protein |  |  | ↑ |
| A0A0A0L935 | Uncharacterized protein |  |  | ↑ |
| A0A0A0K852 | Uncharacterized protein |  |  | ↑ |
| A0A0A0KF02 | Uncharacterized protein |  |  | ↑ |
| A0A0A0KN62 | Uncharacterized protein | - | - | ↑ |
| A0A0A0LRG9 | Uncharacterized protein | - | - | ↑ |
| A0A0A0L505 | MFP1 attachment factor 1 | - | - | ↑ |
| A0A0A0L7A7 | PRA1 family protein | - | - | ↑ |
| A0A0A0LJF9 | Uncharacterized protein | - | - | ↑ |
| A0A0A0LBK8 | Uncharacterized protein |  |  | ↑ |
| A0A0A0LN29 | Uncharacterized protein |  |  | ↑ |
| A0A0A0K9R0 | Uncharacterized protein |  |  | ↑ |
| A0A0A0KA91 | Cytochrome P450 | - | - | ↑ |
| A0A0A0K985 | Uncharacterized protein | - | - | ↑ |
| A0A0A0K4Y1 | Uncharacterized protein |  |  | ↑ |
| A0A0A0LYE7 | Uncharacterized protein |  |  | ↑ |
| A0A0A0LIB1 | Uncharacterized protein |  |  | ↑ |
| A0A0A0LPY4 | Nascent polypeptide-associated complex subunit beta | - | - | ↑ |
| A0A0A0LIJ3 | Uncharacterized protein | - | - | ↑ |
| A0A0A0K9R6 | Uncharacterized protein | - | - | ↑ |
| A0A0A0KGJ2 | DNA ligase | - | - | ↑ |
| A0A0A0LV83 | Uncharacterized protein | - | - | ↑ |
| A0A0A0K9G2 | Uncharacterized protein | - | - | ↑ |
| A0A0A0KZG2 | Pectate lyase | - | - | ↑ |
| A0A0A0KNN2 | Uncharacterized protein | - | - | ↑ |
| A0A0A0LQ38 | Uncharacterized protein | - | - | ↑ |
| A0A0A0KV62 | Uncharacterized protein | - | - | ↑ |
| A0A0A0L4C8 | Uncharacterized protein | - | - | ↑ |
| A0A0A0LUC6 | Uncharacterized protein | - | - | ↑ |
| Q40559 | Peroxidase | - | - | ↑ |
| A0A0A0L1W2 | Uncharacterized protein | - | - | ↑ |
| A0A0A0L734 | Uncharacterized protein | - | - | ↑ |
| A0A0A0K431 | Uncharacterized protein | - | - | ↑ |
| A0A0A0LZJ5 | Uncharacterized protein | - | - | ↑ |
| A0A0A0L0G6 | Uncharacterized protein | - | - | ↑ |
| A0A0A0KQ10 | Uncharacterized protein | - | - | ↑ |
| A0A0A0KX06 | Uncharacterized protein | - | - | ↑ |
| A0A0A0KNW6 | Uncharacterized protein |  |  | ↑ |
| A0A0A0KGI0 | Uncharacterized protein |  |  | ↑ |
| A0A0A0LI17 | Uncharacterized protein | - | - | ↑ |
| A0A0A0L5W2 | Uncharacterized protein | - | - | ↑ |
| A0A0A0LJG3 | Rac-type small GTP-binding protein | - | - | ↑ |
| A0A0A0LGI7 | Uncharacterized protein | - | - | ↑ |
| A0A0A0KAR5 | Uncharacterized protein | - | - | ↑ |
| A0A0A0LW19 | Uncharacterized protein | - | - | ↑ |
| A0A0A0LGM7 | Uncharacterized protein | - | - | ↑ |
| A0A0A0LG03 | Uncharacterized protein | - | - | ↑ |
| A0A0A0K2X7 | Uncharacterized protein |  |  |  |
| A0A0A0KDA2 | Uncharacterized protein | - | - | ↑ |
| A0A0A0LHS9 | Uncharacterized protein | - | - | ↑ |
| A0A0A0KHH9 | Uncharacterized protein | - | - | ↑ |
| A0A0A0KDT1 | Autophagy-related protein 3 | - | - | ↑ |
| A0A0A0LDU9 | Uncharacterized protein | - | - | ↑ |
| A0A0A0LLE8 | Uncharacterized protein | - | - | ↑ |
| A0A0A0LCK0 | DNA helicase | - | - | ↑ |
| A0A0A0KIB2 | Uncharacterized protein | - | - | ↑ |
| A0A0A0KCC5 | Uncharacterized protein | - | - | ↑ |
| A0A0A0LBC6 | Uncharacterized protein | - | - | ↑ |
| A0A0A0KVH9 | Uncharacterized protein | - | - | ↑ |
| A0A0A0KQ14 | Uncharacterized protein | - | - | ↑ |
| A0A0A0KF74 | Uncharacterized protein | - | - | ↓ |
| A0A0A0L0U5 | Chlorophyll a-b binding protein, chloroplastic | - | - | ↓ |
| A0A0A0KZI0 | Uncharacterized protein | - | - | ↓ |
| A0A0A0K913 | Uncharacterized protein | - | - | ↓ |
| A0A0A0LBP0 | Uncharacterized protein | - | - | ↓ |
| A0A0A0L4Q9 | Uncharacterized protein | - | - | ↓ |
| A0A0A0KKC3 | Threonine dehydratase | - | - | ↓ |
| A0A0A0KST8 | Uncharacterized protein | - | - | ↓ |
| A0A0A0LHM2 | Uncharacterized protein | - | - | ↓ |
| A0A0A0L507 | Uncharacterized protein | - | - | ↓ |
| A0A0A0L882 | Uncharacterized protein | - | - | ↓ |
| A0A0A0LS25 | Tonoplast intrinsic protein | - | - | ↓ |
| A0A0A0KFP5 | Uncharacterized protein | - | - | ↓ |
| A0A0A0L6P5 | Uncharacterized protein | - | - | ↓ |
| A0A0A0KGQ9 | Uncharacterized protein | - | - | ↓ |
| A0A0A0LCI6 | Uncharacterized protein | - | - | ↓ |
| A0A0A0K6G7 | Uncharacterized protein | - | - | ↓ |
| A0A0A0L919 | Uncharacterized protein | - | - | ↓ |
| A0A0A0K6C4 | Uncharacterized protein | - | - | ↓ |
| A0A0A0LQ44 | Uncharacterized protein | - | - | ↓ |
| A0A0A0KQQ1 | Uncharacterized protein | - | - | ↓ |
| A0A0A0LX69 | V-type proton ATPase subunit F | - | - | ↓ |
| A0A0A0KKP5 | Ribosomal protein L15 | - | - | ↓ |
| A0A0A0LGC9 | Uncharacterized protein | - | - | ↓ |
| A0A0A0KB82 | Uncharacterized protein | - | - | ↓ |
| A0A0A0KWD0 | Uncharacterized protein | - | - | ↓ |
| A0A0A0KQH3 | Uncharacterized protein | - | - | ↓ |
| A0A0A0M0U7 | Uncharacterized protein | - | - | ↓ |
| A0A0A0LW18 | Uncharacterized protein | - | - | ↓ |
| A0A0A0KL77 | Uncharacterized protein | - | - | ↓ |
| A0A0A0KJJ0 | Uncharacterized protein | - | - | ↓ |
| A0A0A0K773 | Phloem protein 2 | - | - | ↓ |
| A0A0A0LLA2 | Uncharacterized protein | - | - | ↓ |
| A0A0A0KHX8 | Uncharacterized protein | - | - | ↓ |
| A0A0A0K762 | Glycosyltransferase | - | - | ↓ |
| A0A0A0KG08 | Diacylglycerol kinase | - | - | ↓ |
| A0A0A0LT61 | Uncharacterized protein | - | - | ↓ |
| A0A0A0LT92 | Uncharacterized protein | - | - | ↓ |
| A0A0A0LB58 | Uncharacterized protein | - | - | ↓ |
| A0A0A0KK13 | Uncharacterized protein | - | - | ↓ |
| A0A0A0KI85 | Uncharacterized protein | - | - | ↓ |
| A0A0A0KV98 | Chlorophyll a-b binding protein, chloroplastic | - | - | ↓ |
| A0A0A0K3W6 | Phloem filament protein | - | - | ↓ |
| A0A0A0KBK4 | Uncharacterized protein | - | - | ↓ |
