# Supplementary material for: Proteomic analysis reveals key proteins involved in ethylene-induced adventitious root development in cucumber (Cucumis sativus L.)
Source: PeerJ. 2021 Apr 6;9:e10887. doi: 10.7717/peerj.10887 (PMC8034359; doi:10.7717/peerj.10887)
Supplement: Supplemental Information 6 [file peerj-09-10887-s006.zip › 6.23-Blot pictures.doc]

**The original images of western blots**

actin

AtpA

SAM1-4-S-adenosylmethionine syn

CK-0h CK-12h CK-24h CK-48h ETH-0h ETH-12h ETH-24h ETH-48h
